# Measurement of $WW/WZ \rightarrow \ell \nu q q^{\prime }$ production with the hadronically decaying boson reconstructed as one or two jets in *pp* collisions at $\sqrt{s} =8~\text {TeV}$ with ATLAS, and constraints on anomalous gauge couplings

**DOI:** 10.1140/epjc/s10052-017-5084-2

**Published:** 2017-08-20

**Authors:** M. Aaboud, G. Aad, B. Abbott, O. Abdinov, B. Abeloos, S. H. Abidi, O. S. AbouZeid, N. L. Abraham, H. Abramowicz, H. Abreu, R. Abreu, Y. Abulaiti, B. S. Acharya, S. Adachi, L. Adamczyk, J. Adelman, M. Adersberger, T. Adye, A. A. Affolder, T. Agatonovic-Jovin, C. Agheorghiesei, J. A. Aguilar-Saavedra, S. P. Ahlen, F. Ahmadov, G. Aielli, S. Akatsuka, H. Akerstedt, T. P. A. Åkesson, E. Akilli, A. V. Akimov, G. L. Alberghi, J. Albert, P. Albicocco, M. J. Alconada Verzini, S. C. Alderweireldt, M. Aleksa, I. N. Aleksandrov, C. Alexa, G. Alexander, T. Alexopoulos, M. Alhroob, B. Ali, M. Aliev, G. Alimonti, J. Alison, S. P. Alkire, B. M. M. Allbrooke, B. W. Allen, P. P. Allport, A. Aloisio, A. Alonso, F. Alonso, C. Alpigiani, A. A. Alshehri, M. I. Alstaty, B. Alvarez Gonzalez, D. Álvarez Piqueras, M. G. Alviggi, B. T. Amadio, Y. Amaral Coutinho, C. Amelung, D. Amidei, S. P. Amor Dos Santos, A. Amorim, S. Amoroso, G. Amundsen, C. Anastopoulos, L. S. Ancu, N. Andari, T. Andeen, C. F. Anders, J. K. Anders, K. J. Anderson, A. Andreazza, V. Andrei, S. Angelidakis, I. Angelozzi, A. Angerami, A. V. Anisenkov, N. Anjos, A. Annovi, C. Antel, M. Antonelli, A. Antonov, D. J. Antrim, F. Anulli, M. Aoki, L. Aperio Bella, G. Arabidze, Y. Arai, J. P. Araque, V. Araujo Ferraz, A. T. H. Arce, R. E. Ardell, F. A. Arduh, J-F. Arguin, S. Argyropoulos, M. Arik, A. J. Armbruster, L. J. Armitage, O. Arnaez, H. Arnold, M. Arratia, O. Arslan, A. Artamonov, G. Artoni, S. Artz, S. Asai, N. Asbah, A. Ashkenazi, L. Asquith, K. Assamagan, R. Astalos, M. Atkinson, N. B. Atlay, K. Augsten, G. Avolio, B. Axen, M. K. Ayoub, G. Azuelos, A. E. Baas, M. J. Baca, H. Bachacou, K. Bachas, M. Backes, M. Backhaus, P. Bagnaia, M. Bahmani, H. Bahrasemani, J. T. Baines, M. Bajic, O. K. Baker, E. M. Baldin, P. Balek, F. Balli, W. K. Balunas, E. Banas, A. Bandyopadhyay, Sw. Banerjee, A. A. E. Bannoura, L. Barak, E. L. Barberio, D. Barberis, M. Barbero, T. Barillari, M-S Barisits, J. T. Barkeloo, T. Barklow, N. Barlow, S. L. Barnes, B. M. Barnett, R. M. Barnett, Z. Barnovska-Blenessy, A. Baroncelli, G. Barone, A. J. Barr, L. Barranco Navarro, F. Barreiro, J. Barreiro Guimarães da Costa, R. Bartoldus, A. E. Barton, P. Bartos, A. Basalaev, A. Bassalat, R. L. Bates, S. J. Batista, J. R. Batley, M. Battaglia, M. Bauce, F. Bauer, H. S. Bawa, J. B. Beacham, M. D. Beattie, T. Beau, P. H. Beauchemin, P. Bechtle, H. P. Beck, H. C. Beck, K. Becker, M. Becker, M. Beckingham, C. Becot, A. J. Beddall, A. Beddall, V. A. Bednyakov, M. Bedognetti, C. P. Bee, T. A. Beermann, M. Begalli, M. Begel, J. K. Behr, A. S. Bell, G. Bella, L. Bellagamba, A. Bellerive, M. Bellomo, K. Belotskiy, O. Beltramello, N. L. Belyaev, O. Benary, D. Benchekroun, M. Bender, K. Bendtz, N. Benekos, Y. Benhammou, E. Benhar Noccioli, J. Benitez, D. P. Benjamin, M. Benoit, J. R. Bensinger, S. Bentvelsen, L. Beresford, M. Beretta, D. Berge, E. Bergeaas Kuutmann, N. Berger, J. Beringer, S. Berlendis, N. R. Bernard, G. Bernardi, C. Bernius, F. U. Bernlochner, T. Berry, P. Berta, C. Bertella, G. Bertoli, F. Bertolucci, I. A. Bertram, C. Bertsche, D. Bertsche, G. J. Besjes, O. Bessidskaia Bylund, M. Bessner, N. Besson, C. Betancourt, A. Bethani, S. Bethke, A. J. Bevan, J. Beyer, R. M. Bianchi, O. Biebel, D. Biedermann, R. Bielski, K. Bierwagen, N. V. Biesuz, M. Biglietti, T. R. V. Billoud, H. Bilokon, M. Bindi, A. Bingul, C. Bini, S. Biondi, T. Bisanz, C. Bittrich, D. M. Bjergaard, C. W. Black, J. E. Black, K. M. Black, R. E. Blair, T. Blazek, I. Bloch, C. Blocker, A. Blue, W. Blum, U. Blumenschein, S. Blunier, G. J. Bobbink, V. S. Bobrovnikov, S. S. Bocchetta, A. Bocci, C. Bock, M. Boehler, D. Boerner, D. Bogavac, A. G. Bogdanchikov, C. Bohm, V. Boisvert, P. Bokan, T. Bold, A. S. Boldyrev, A. E. Bolz, M. Bomben, M. Bona, M. Boonekamp, A. Borisov, G. Borissov, J. Bortfeldt, D. Bortoletto, V. Bortolotto, D. Boscherini, M. Bosman, J. D. Bossio Sola, J. Boudreau, J. Bouffard, E. V. Bouhova-Thacker, D. Boumediene, C. Bourdarios, S. K. Boutle, A. Boveia, J. Boyd, I. R. Boyko, J. Bracinik, A. Brandt, G. Brandt, O. Brandt, U. Bratzler, B. Brau, J. E. Brau, W. D. Breaden Madden, K. Brendlinger, A. J. Brennan, L. Brenner, R. Brenner, S. Bressler, D. L. Briglin, T. M. Bristow, D. Britton, D. Britzger, F. M. Brochu, I. Brock, R. Brock, G. Brooijmans, T. Brooks, W. K. Brooks, J. Brosamer, E. Brost, J. H Broughton, P. A. Bruckman de Renstrom, D. Bruncko, A. Bruni, G. Bruni, L. S. Bruni, B H Brunt, M. Bruschi, N. Bruscino, P. Bryant, L. Bryngemark, T. Buanes, Q. Buat, P. Buchholz, A. G. Buckley, I. A. Budagov, F. Buehrer, M. K. Bugge, O. Bulekov, D. Bullock, T. J. Burch, S. Burdin, C. D. Burgard, A. M. Burger, B. Burghgrave, K. Burka, S. Burke, I. Burmeister, J. T. P. Burr, E. Busato, D. Büscher, V. Büscher, P. Bussey, J. M. Butler, C. M. Buttar, J. M. Butterworth, P. Butti, W. Buttinger, A. Buzatu, A. R. Buzykaev, S. Cabrera Urbán, D. Caforio, V. M. Cairo, O. Cakir, N. Calace, P. Calafiura, A. Calandri, G. Calderini, P. Calfayan, G. Callea, L. P. Caloba, S. Calvente Lopez, D. Calvet, S. Calvet, T. P. Calvet, M. Calvetti, R. Camacho Toro, S. Camarda, P. Camarri, D. Cameron, R. Caminal Armadans, C. Camincher, S. Campana, M. Campanelli, A. Camplani, A. Campoverde, V. Canale, M. Cano Bret, J. Cantero, T. Cao, M. D. M. Capeans Garrido, I. Caprini, M. Caprini, M. Capua, R. M. Carbone, R. Cardarelli, F. Cardillo, I. Carli, T. Carli, G. Carlino, B. T. Carlson, L. Carminati, R. M. D. Carney, S. Caron, E. Carquin, S. Carrá, G. D. Carrillo-Montoya, J. Carvalho, D. Casadei, M. P. Casado, M. Casolino, D. W. Casper, R. Castelijn, V. Castillo Gimenez, N. F. Castro, A. Catinaccio, J. R. Catmore, A. Cattai, J. Caudron, V. Cavaliere, E. Cavallaro, D. Cavalli, M. Cavalli-Sforza, V. Cavasinni, E. Celebi, F. Ceradini, L. Cerda Alberich, A. S. Cerqueira, A. Cerri, L. Cerrito, F. Cerutti, A. Cervelli, S. A. Cetin, A. Chafaq, D. Chakraborty, S. K. Chan, W. S. Chan, Y. L. Chan, P. Chang, J. D. Chapman, D. G. Charlton, C. C. Chau, C. A. Chavez Barajas, S. Che, S. Cheatham, A. Chegwidden, S. Chekanov, S. V. Chekulaev, G. A. Chelkov, M. A. Chelstowska, C. Chen, H. Chen, J. Chen, S. Chen, S. Chen, X. Chen, Y. Chen, H. C. Cheng, H. J. Cheng, A. Cheplakov, E. Cheremushkina, R. Cherkaoui El Moursli, E. Cheu, K. Cheung, L. Chevalier, V. Chiarella, G. Chiarelli, G. Chiodini, A. S. Chisholm, A. Chitan, Y. H. Chiu, M. V. Chizhov, K. Choi, A. R. Chomont, S. Chouridou, Y. S. Chow, V. Christodoulou, M. C. Chu, J. Chudoba, A. J. Chuinard, J. J. Chwastowski, L. Chytka, A. K. Ciftci, D. Cinca, V. Cindro, I. A. Cioara, C. Ciocca, A. Ciocio, F. Cirotto, Z. H. Citron, M. Citterio, M. Ciubancan, A. Clark, B. L. Clark, M. R. Clark, P. J. Clark, R. N. Clarke, C. Clement, Y. Coadou, M. Cobal, A. Coccaro, J. Cochran, L. Colasurdo, B. Cole, A. P. Colijn, J. Collot, T. Colombo, P. Conde Muiño, E. Coniavitis, S. H. Connell, I. A. Connelly, S. Constantinescu, G. Conti, F. Conventi, M. Cooke, A. M. Cooper-Sarkar, F. Cormier, K. J. R. Cormier, M. Corradi, F. Corriveau, A. Cortes-Gonzalez, G. Cortiana, G. Costa, M. J. Costa, D. Costanzo, G. Cottin, G. Cowan, B. E. Cox, K. Cranmer, S. J. Crawley, R. A. Creager, G. Cree, S. Crépé-Renaudin, F. Crescioli, W. A. Cribbs, M. Cristinziani, V. Croft, G. Crosetti, A. Cueto, T. Cuhadar Donszelmann, A. R. Cukierman, J. Cummings, M. Curatolo, J. Cúth, S. Czekierda, P. Czodrowski, G. D’amen, S. D’Auria, L. D’eramo, M. D’Onofrio, M. J. Da Cunha Sargedas De Sousa, C. Da Via, W. Dabrowski, T. Dado, T. Dai, O. Dale, F. Dallaire, C. Dallapiccola, M. Dam, J. R. Dandoy, M. F. Daneri, N. P. Dang, A. C. Daniells, N. S. Dann, M. Danninger, M. Dano Hoffmann, V. Dao, G. Darbo, S. Darmora, J. Dassoulas, A. Dattagupta, T. Daubney, W. Davey, C. David, T. Davidek, D. R. Davis, P. Davison, E. Dawe, I. Dawson, K. De, R. de Asmundis, A. De Benedetti, S. De Castro, S. De Cecco, N. De Groot, P. de Jong, H. De la Torre, F. De Lorenzi, A. De Maria, D. De Pedis, A. De Salvo, U. De Sanctis, A. De Santo, K. De Vasconcelos Corga, J. B. De Vivie De Regie, W. J. Dearnaley, R. Debbe, C. Debenedetti, D. V. Dedovich, N. Dehghanian, I. Deigaard, M. Del Gaudio, J. Del Peso, D. Delgove, F. Deliot, C. M. Delitzsch, A. Dell’Acqua, L. Dell’Asta, M. Dell’Orso, M. Della Pietra, D. della Volpe, M. Delmastro, C. Delporte, P. A. Delsart, D. A. DeMarco, S. Demers, M. Demichev, A. Demilly, S. P. Denisov, D. Denysiuk, D. Derendarz, J. E. Derkaoui, F. Derue, P. Dervan, K. Desch, C. Deterre, K. Dette, M. R. Devesa, P. O. Deviveiros, A. Dewhurst, S. Dhaliwal, F. A. Di Bello, A. Di Ciaccio, L. Di Ciaccio, W. K. Di Clemente, C. Di Donato, A. Di Girolamo, B. Di Girolamo, B. Di Micco, R. Di Nardo, K. F. Di Petrillo, A. Di Simone, R. Di Sipio, D. Di Valentino, C. Diaconu, M. Diamond, F. A. Dias, M. A. Diaz, E. B. Diehl, J. Dietrich, S. Díez Cornell, A. Dimitrievska, J. Dingfelder, P. Dita, S. Dita, F. Dittus, F. Djama, T. Djobava, J. I. Djuvsland, M. A. B. do Vale, D. Dobos, M. Dobre, C. Doglioni, J. Dolejsi, Z. Dolezal, M. Donadelli, S. Donati, P. Dondero, J. Donini, J. Dopke, A. Doria, M. T. Dova, A. T. Doyle, E. Drechsler, M. Dris, Y. Du, J. Duarte-Campderros, A. Dubreuil, E. Duchovni, G. Duckeck, A. Ducourthial, O. A. Ducu, D. Duda, A. Dudarev, A. Chr. Dudder, E. M. Duffield, L. Duflot, M. Dührssen, M. Dumancic, A. E. Dumitriu, A. K. Duncan, M. Dunford, H. Duran Yildiz, M. Düren, A. Durglishvili, D. Duschinger, B. Dutta, D. Duvnjak, M. Dyndal, B. S. Dziedzic, C. Eckardt, K. M. Ecker, R. C. Edgar, T. Eifert, G. Eigen, K. Einsweiler, T. Ekelof, M. El Kacimi, R. El Kosseifi, V. Ellajosyula, M. Ellert, S. Elles, F. Ellinghaus, A. A. Elliot, N. Ellis, J. Elmsheuser, M. Elsing, D. Emeliyanov, Y. Enari, O. C. Endner, J. S. Ennis, J. Erdmann, A. Ereditato, M. Ernst, S. Errede, M. Escalier, C. Escobar, B. Esposito, O. Estrada Pastor, A. I. Etienvre, E. Etzion, H. Evans, A. Ezhilov, M. Ezzi, F. Fabbri, L. Fabbri, V. Fabiani, G. Facini, R. M. Fakhrutdinov, S. Falciano, R. J. Falla, J. Faltova, Y. Fang, M. Fanti, A. Farbin, A. Farilla, C. Farina, E. M. Farina, T. Farooque, S. Farrell, S. M. Farrington, P. Farthouat, F. Fassi, P. Fassnacht, D. Fassouliotis, M. Faucci Giannelli, A. Favareto, W. J. Fawcett, L. Fayard, O. L. Fedin, W. Fedorko, S. Feigl, L. Feligioni, C. Feng, E. J. Feng, H. Feng, M. J. Fenton, A. B. Fenyuk, L. Feremenga, P. Fernandez Martinez, S. Fernandez Perez, J. Ferrando, A. Ferrari, P. Ferrari, R. Ferrari, D. E. Ferreira de Lima, A. Ferrer, D. Ferrere, C. Ferretti, F. Fiedler, A. Filipčič, M. Filipuzzi, F. Filthaut, M. Fincke-Keeler, K. D. Finelli, M. C. N. Fiolhais, L. Fiorini, A. Fischer, C. Fischer, J. Fischer, W. C. Fisher, N. Flaschel, I. Fleck, P. Fleischmann, R. R. M. Fletcher, T. Flick, B. M. Flierl, L. R. Flores Castillo, M. J. Flowerdew, G. T. Forcolin, A. Formica, F. A. Förster, A. Forti, A. G. Foster, D. Fournier, H. Fox, S. Fracchia, P. Francavilla, M. Franchini, S. Franchino, D. Francis, L. Franconi, M. Franklin, M. Frate, M. Fraternali, D. Freeborn, S. M. Fressard-Batraneanu, B. Freund, D. Froidevaux, J. A. Frost, C. Fukunaga, T. Fusayasu, J. Fuster, C. Gabaldon, O. Gabizon, A. Gabrielli, A. Gabrielli, G. P. Gach, S. Gadatsch, S. Gadomski, G. Gagliardi, L. G. Gagnon, C. Galea, B. Galhardo, E. J. Gallas, B. J. Gallop, P. Gallus, G. Galster, K. K. Gan, S. Ganguly, Y. Gao, Y. S. Gao, F. M. Garay Walls, C. García, J. E. García Navarro, J. A. García Pascual, M. Garcia-Sciveres, R. W. Gardner, N. Garelli, V. Garonne, A. Gascon Bravo, K. Gasnikova, C. Gatti, A. Gaudiello, G. Gaudio, I. L. Gavrilenko, C. Gay, G. Gaycken, E. N. Gazis, C. N. P. Gee, J. Geisen, M. Geisen, M. P. Geisler, K. Gellerstedt, C. Gemme, M. H. Genest, C. Geng, S. Gentile, C. Gentsos, S. George, D. Gerbaudo, A. Gershon, G. Geßner, S. Ghasemi, M. Ghneimat, B. Giacobbe, S. Giagu, N. Giangiacomi, P. Giannetti, S. M. Gibson, M. Gignac, M. Gilchriese, D. Gillberg, G. Gilles, D. M. Gingrich, N. Giokaris, M. P. Giordani, F. M. Giorgi, P. F. Giraud, P. Giromini, G. Giugliarelli, D. Giugni, F. Giuli, C. Giuliani, M. Giulini, B. K. Gjelsten, S. Gkaitatzis, I. Gkialas, E. L. Gkougkousis, P. Gkountoumis, L. K. Gladilin, C. Glasman, J. Glatzer, P. C. F. Glaysher, A. Glazov, M. Goblirsch-Kolb, J. Godlewski, S. Goldfarb, T. Golling, D. Golubkov, A. Gomes, R. Gonçalo, R. Goncalves Gama, J. Goncalves Pinto Firmino Da Costa, G. Gonella, L. Gonella, A. Gongadze, S. González de la Hoz, S. Gonzalez-Sevilla, L. Goossens, P. A. Gorbounov, H. A. Gordon, I. Gorelov, B. Gorini, E. Gorini, A. Gorišek, A. T. Goshaw, C. Gössling, M. I. Gostkin, C. A. Gottardo, C. R. Goudet, D. Goujdami, A. G. Goussiou, N. Govender, E. Gozani, L. Graber, I. Grabowska-Bold, P. O. J. Gradin, J. Gramling, E. Gramstad, S. Grancagnolo, V. Gratchev, P. M. Gravila, C. Gray, H. M. Gray, Z. D. Greenwood, C. Grefe, K. Gregersen, I. M. Gregor, P. Grenier, K. Grevtsov, J. Griffiths, A. A. Grillo, K. Grimm, S. Grinstein, Ph. Gris, J.-F. Grivaz, S. Groh, E. Gross, J. Grosse-Knetter, G. C. Grossi, Z. J. Grout, A. Grummer, L. Guan, W. Guan, J. Guenther, F. Guescini, D. Guest, O. Gueta, B. Gui, E. Guido, T. Guillemin, S. Guindon, U. Gul, C. Gumpert, J. Guo, W. Guo, Y. Guo, R. Gupta, S. Gupta, G. Gustavino, P. Gutierrez, N. G. Gutierrez Ortiz, C. Gutschow, C. Guyot, M. P. Guzik, C. Gwenlan, C. B. Gwilliam, A. Haas, C. Haber, H. K. Hadavand, N. Haddad, A. Hadef, S. Hageböck, M. Hagihara, H. Hakobyan, M. Haleem, J. Haley, G. Halladjian, G. D. Hallewell, K. Hamacher, P. Hamal, K. Hamano, A. Hamilton, G. N. Hamity, P. G. Hamnett, L. Han, S. Han, K. Hanagaki, K. Hanawa, M. Hance, B. Haney, P. Hanke, J. B. Hansen, J. D. Hansen, M. C. Hansen, P. H. Hansen, K. Hara, A. S. Hard, T. Harenberg, F. Hariri, S. Harkusha, R. D. Harrington, P. F. Harrison, N. M. Hartmann, M. Hasegawa, Y. Hasegawa, A. Hasib, S. Hassani, S. Haug, R. Hauser, L. Hauswald, L. B. Havener, M. Havranek, C. M. Hawkes, R. J. Hawkings, D. Hayakawa, D. Hayden, C. P. Hays, J. M. Hays, H. S. Hayward, S. J. Haywood, S. J. Head, T. Heck, V. Hedberg, L. Heelan, S. Heer, K. K. Heidegger, S. Heim, T. Heim, B. Heinemann, J. J. Heinrich, L. Heinrich, C. Heinz, J. Hejbal, L. Helary, A. Held, S. Hellman, C. Helsens, R. C. W. Henderson, Y. Heng, S. Henkelmann, A. M. Henriques Correia, S. Henrot-Versille, G. H. Herbert, H. Herde, V. Herget, Y. Hernández Jiménez, H. Herr, G. Herten, R. Hertenberger, L. Hervas, T. C. Herwig, G. G. Hesketh, N. P. Hessey, J. W. Hetherly, S. Higashino, E. Higón-Rodriguez, K. Hildebrand, E. Hill, J. C. Hill, K. H. Hiller, S. J. Hillier, M. Hils, I. Hinchliffe, M. Hirose, D. Hirschbuehl, B. Hiti, O. Hladik, X. Hoad, J. Hobbs, N. Hod, M. C. Hodgkinson, P. Hodgson, A. Hoecker, M. R. Hoeferkamp, F. Hoenig, D. Hohn, T. R. Holmes, M. Homann, S. Honda, T. Honda, T. M. Hong, B. H. Hooberman, W. H. Hopkins, Y. Horii, A. J. Horton, J-Y. Hostachy, S. Hou, A. Hoummada, J. Howarth, J. Hoya, M. Hrabovsky, J. Hrdinka, I. Hristova, J. Hrivnac, T. Hryn’ova, A. Hrynevich, P. J. Hsu, S.-C. Hsu, Q. Hu, S. Hu, Y. Huang, Z. Hubacek, F. Hubaut, F. Huegging, T. B. Huffman, E. W. Hughes, G. Hughes, M. Huhtinen, P. Huo, N. Huseynov, J. Huston, J. Huth, G. Iacobucci, G. Iakovidis, I. Ibragimov, L. Iconomidou-Fayard, Z. Idrissi, P. Iengo, O. Igonkina, T. Iizawa, Y. Ikegami, M. Ikeno, Y. Ilchenko, D. Iliadis, N. Ilic, G. Introzzi, P. Ioannou, M. Iodice, K. Iordanidou, V. Ippolito, M. F. Isacson, N. Ishijima, M. Ishino, M. Ishitsuka, C. Issever, S. Istin, F. Ito, J. M. Iturbe Ponce, R. Iuppa, H. Iwasaki, J. M. Izen, V. Izzo, S. Jabbar, P. Jackson, R. M. Jacobs, V. Jain, K. B. Jakobi, K. Jakobs, S. Jakobsen, T. Jakoubek, D. O. Jamin, D. K. Jana, R. Jansky, J. Janssen, M. Janus, P. A. Janus, G. Jarlskog, N. Javadov, T. Javůrek, M. Javurkova, F. Jeanneau, L. Jeanty, J. Jejelava, A. Jelinskas, P. Jenni, C. Jeske, S. Jézéquel, H. Ji, J. Jia, H. Jiang, Y. Jiang, Z. Jiang, S. Jiggins, J. Jimenez Pena, S. Jin, A. Jinaru, O. Jinnouchi, H. Jivan, P. Johansson, K. A. Johns, C. A. Johnson, W. J. Johnson, K. Jon-And, R. W. L. Jones, S. D. Jones, S. Jones, T. J. Jones, J. Jongmanns, P. M. Jorge, J. Jovicevic, X. Ju, A. Juste Rozas, M. K. Köhler, A. Kaczmarska, M. Kado, H. Kagan, M. Kagan, S. J. Kahn, T. Kaji, E. Kajomovitz, C. W. Kalderon, A. Kaluza, S. Kama, A. Kamenshchikov, N. Kanaya, L. Kanjir, V. A. Kantserov, J. Kanzaki, B. Kaplan, L. S. Kaplan, D. Kar, K. Karakostas, N. Karastathis, M. J. Kareem, E. Karentzos, S. N. Karpov, Z. M. Karpova, K. Karthik, V. Kartvelishvili, A. N. Karyukhin, K. Kasahara, L. Kashif, R. D. Kass, A. Kastanas, Y. Kataoka, C. Kato, A. Katre, J. Katzy, K. Kawade, K. Kawagoe, T. Kawamoto, G. Kawamura, E. F. Kay, V. F. Kazanin, R. Keeler, R. Kehoe, J. S. Keller, E. Kellermann, J. J. Kempster, J Kendrick, H. Keoshkerian, O. Kepka, B. P. Kerševan, S. Kersten, R. A. Keyes, M. Khader, F. Khalil-zada, A. Khanov, A. G. Kharlamov, T. Kharlamova, A. Khodinov, T. J. Khoo, V. Khovanskiy, E. Khramov, J. Khubua, S. Kido, C. R. Kilby, H. Y. Kim, S. H. Kim, Y. K. Kim, N. Kimura, O. M. Kind, B. T. King, D. Kirchmeier, J. Kirk, A. E. Kiryunin, T. Kishimoto, D. Kisielewska, V. Kitali, K. Kiuchi, O. Kivernyk, E. Kladiva, T. Klapdor-Kleingrothaus, M. H. Klein, M. Klein, U. Klein, K. Kleinknecht, P. Klimek, A. Klimentov, R. Klingenberg, T. Klingl, T. Klioutchnikova, E.-E. Kluge, P. Kluit, S. Kluth, E. Kneringer, E. B. F. G. Knoops, A. Knue, A. Kobayashi, D. Kobayashi, T. Kobayashi, M. Kobel, M. Kocian, P. Kodys, T. Koffas, E. Koffeman, N. M. Köhler, T. Koi, M. Kolb, I. Koletsou, A. A. Komar, Y. Komori, T. Kondo, N. Kondrashova, K. Köneke, A. C. König, T. Kono, R. Konoplich, N. Konstantinidis, R. Kopeliansky, S. Koperny, A. K. Kopp, K. Korcyl, K. Kordas, A. Korn, A. A. Korol, I. Korolkov, E. V. Korolkova, O. Kortner, S. Kortner, T. Kosek, V. V. Kostyukhin, A. Kotwal, A. Koulouris, A. Kourkoumeli-Charalampidi, C. Kourkoumelis, E. Kourlitis, V. Kouskoura, A. B. Kowalewska, R. Kowalewski, T. Z. Kowalski, C. Kozakai, W. Kozanecki, A. S. Kozhin, V. A. Kramarenko, G. Kramberger, D. Krasnopevtsev, M. W. Krasny, A. Krasznahorkay, D. Krauss, J. A. Kremer, J. Kretzschmar, K. Kreutzfeldt, P. Krieger, K. Krizka, K. Kroeninger, H. Kroha, J. Kroll, J. Kroll, J. Kroseberg, J. Krstic, U. Kruchonak, H. Krüger, N. Krumnack, M. C. Kruse, T. Kubota, H. Kucuk, S. Kuday, J. T. Kuechler, S. Kuehn, A. Kugel, F. Kuger, T. Kuhl, V. Kukhtin, R. Kukla, Y. Kulchitsky, S. Kuleshov, Y. P. Kulinich, M. Kuna, T. Kunigo, A. Kupco, T. Kupfer, O. Kuprash, H. Kurashige, L. L. Kurchaninov, Y. A. Kurochkin, M. G. Kurth, V. Kus, E. S. Kuwertz, M. Kuze, J. Kvita, T. Kwan, D. Kyriazopoulos, A. La Rosa, J. L. La Rosa Navarro, L. La Rotonda, F. La Ruffa, C. Lacasta, F. Lacava, J. Lacey, H. Lacker, D. Lacour, E. Ladygin, R. Lafaye, B. Laforge, T. Lagouri, S. Lai, S. Lammers, W. Lampl, E. Lançon, U. Landgraf, M. P. J. Landon, M. C. Lanfermann, V. S. Lang, J. C. Lange, R. J. Langenberg, A. J. Lankford, F. Lanni, K. Lantzsch, A. Lanza, A. Lapertosa, S. Laplace, J. F. Laporte, T. Lari, F. Lasagni Manghi, M. Lassnig, P. Laurelli, W. Lavrijsen, A. T. Law, P. Laycock, T. Lazovich, M. Lazzaroni, B. Le, O. Le Dortz, E. Le Guirriec, E. P. Le Quilleuc, M. LeBlanc, T. LeCompte, F. Ledroit-Guillon, C. A. Lee, G. R. Lee, S. C. Lee, L. Lee, B. Lefebvre, G. Lefebvre, M. Lefebvre, F. Legger, C. Leggett, G. Lehmann Miotto, X. Lei, W. A. Leight, M. A. L. Leite, R. Leitner, D. Lellouch, B. Lemmer, K. J. C. Leney, T. Lenz, B. Lenzi, R. Leone, S. Leone, C. Leonidopoulos, G. Lerner, C. Leroy, A. A. J. Lesage, C. G. Lester, M. Levchenko, J. Levêque, D. Levin, L. J. Levinson, M. Levy, D. Lewis, B. Li, C.-Q Li, H. Li, L. Li, Q. Li, S. Li, X. Li, Y. Li, Z. Liang, B. Liberti, A. Liblong, K. Lie, J. Liebal, W. Liebig, A. Limosani, S. C. Lin, T. H. Lin, R. A. Linck, B. E. Lindquist, A. E. Lionti, E. Lipeles, A. Lipniacka, M. Lisovyi, T. M. Liss, A. Lister, A. M. Litke, B. Liu, H. Liu, H. Liu, J. K. K. Liu, J. Liu, J. B. Liu, K. Liu, L. Liu, M. Liu, Y. L. Liu, Y. Liu, M. Livan, A. Lleres, J. Llorente Merino, S. L. Lloyd, C. Y. Lo, F. Lo Sterzo, E. M. Lobodzinska, P. Loch, F. K. Loebinger, A. Loesle, K. M. Loew, A. Loginov, T. Lohse, K. Lohwasser, M. Lokajicek, B. A. Long, J. D. Long, R. E. Long, L. Longo, K. A. Looper, J. A. Lopez, D. Lopez Mateos, I. Lopez Paz, A. Lopez Solis, J. Lorenz, N. Lorenzo Martinez, M. Losada, P. J. Lösel, X. Lou, A. Lounis, J. Love, P. A. Love, H. Lu, N. Lu, Y. J. Lu, H. J. Lubatti, C. Luci, A. Lucotte, C. Luedtke, F. Luehring, W. Lukas, L. Luminari, O. Lundberg, B. Lund-Jensen, M. S. Lutz, P. M. Luzi, D. Lynn, R. Lysak, E. Lytken, F. Lyu, V. Lyubushkin, H. Ma, L. L. Ma, Y. Ma, G. Maccarrone, A. Macchiolo, C. M. Macdonald, B. Maček, J. Machado Miguens, D. Madaffari, R. Madar, W. F. Mader, A. Madsen, J. Maeda, S. Maeland, T. Maeno, A. S. Maevskiy, V. Magerl, J. Mahlstedt, C. Maiani, C. Maidantchik, A. A. Maier, T. Maier, A. Maio, O. Majersky, S. Majewski, Y. Makida, N. Makovec, B. Malaescu, Pa. Malecki, V. P. Maleev, F. Malek, U. Mallik, D. Malon, C. Malone, S. Maltezos, S. Malyukov, J. Mamuzic, G. Mancini, I. Mandić, J. Maneira, L. Manhaes de Andrade Filho, J. Manjarres Ramos, K. H. Mankinen, A. Mann, A. Manousos, B. Mansoulie, J. D. Mansour, R. Mantifel, M. Mantoani, S. Manzoni, L. Mapelli, G. Marceca, L. March, L. Marchese, G. Marchiori, M. Marcisovsky, M. Marjanovic, D. E. Marley, F. Marroquim, S. P. Marsden, Z. Marshall, M. U. F Martensson, S. Marti-Garcia, C. B. Martin, T. A. Martin, V. J. Martin, B. Martin dit Latour, M. Martinez, V. I. Martinez Outschoorn, S. Martin-Haugh, V. S. Martoiu, A. C. Martyniuk, A. Marzin, L. Masetti, T. Mashimo, R. Mashinistov, J. Masik, A. L. Maslennikov, L. Massa, P. Mastrandrea, A. Mastroberardino, T. Masubuchi, P. Mättig, J. Maurer, S. J. Maxfield, D. A. Maximov, R. Mazini, I. Maznas, S. M. Mazza, N. C. Mc Fadden, G. Mc Goldrick, S. P. Mc Kee, A. McCarn, R. L. McCarthy, T. G. McCarthy, L. I. McClymont, E. F. McDonald, J. A. Mcfayden, G. Mchedlidze, S. J. McMahon, P. C. McNamara, R. A. McPherson, S. Meehan, T. J. Megy, S. Mehlhase, A. Mehta, T. Meideck, K. Meier, B. Meirose, D. Melini, B. R. Mellado Garcia, J. D. Mellenthin, M. Melo, F. Meloni, A. Melzer, S. B. Menary, L. Meng, X. T. Meng, A. Mengarelli, S. Menke, E. Meoni, S. Mergelmeyer, P. Mermod, L. Merola, C. Meroni, F. S. Merritt, A. Messina, J. Metcalfe, A. S. Mete, C. Meyer, J-P. Meyer, J. Meyer, H. Meyer Zu Theenhausen, F. Miano, R. P. Middleton, S. Miglioranzi, L. Mijović, G. Mikenberg, M. Mikestikova, M. Mikuž, M. Milesi, A. Milic, D. W. Miller, C. Mills, A. Milov, D. A. Milstead, A. A. Minaenko, Y. Minami, I. A. Minashvili, A. I. Mincer, B. Mindur, M. Mineev, Y. Minegishi, Y. Ming, L. M. Mir, K. P. Mistry, T. Mitani, J. Mitrevski, V. A. Mitsou, A. Miucci, P. S. Miyagawa, A. Mizukami, J. U. Mjörnmark, T. Mkrtchyan, M. Mlynarikova, T. Moa, K. Mochizuki, P. Mogg, S. Mohapatra, S. Molander, R. Moles-Valls, R. Monden, M. C. Mondragon, K. Mönig, J. Monk, E. Monnier, A. Montalbano, J. Montejo Berlingen, F. Monticelli, S. Monzani, R. W. Moore, N. Morange, D. Moreno, M. Moreno Llácer, P. Morettini, S. Morgenstern, D. Mori, T. Mori, M. Morii, M. Morinaga, V. Morisbak, A. K. Morley, G. Mornacchi, J. D. Morris, L. Morvaj, P. Moschovakos, M. Mosidze, H. J. Moss, J. Moss, K. Motohashi, R. Mount, E. Mountricha, E. J. W. Moyse, S. Muanza, F. Mueller, J. Mueller, R. S. P. Mueller, D. Muenstermann, P. Mullen, G. A. Mullier, F. J. Munoz Sanchez, W. J. Murray, H. Musheghyan, M. Muškinja, A. G. Myagkov, M. Myska, B. P. Nachman, O. Nackenhorst, K. Nagai, R. Nagai, K. Nagano, Y. Nagasaka, K. Nagata, M. Nagel, E. Nagy, A. M. Nairz, Y. Nakahama, K. Nakamura, T. Nakamura, I. Nakano, R. F. Naranjo Garcia, R. Narayan, D. I. Narrias Villar, I. Naryshkin, T. Naumann, G. Navarro, R. Nayyar, H. A. Neal, P. Yu. Nechaeva, T. J. Neep, A. Negri, M. Negrini, S. Nektarijevic, C. Nellist, A. Nelson, M. E. Nelson, S. Nemecek, P. Nemethy, M. Nessi, M. S. Neubauer, M. Neumann, P. R. Newman, T. Y. Ng, T. Nguyen Manh, R. B. Nickerson, R. Nicolaidou, J. Nielsen, V. Nikolaenko, I. Nikolic-Audit, K. Nikolopoulos, J. K. Nilsen, P. Nilsson, Y. Ninomiya, A. Nisati, N. Nishu, R. Nisius, I. Nitsche, T. Nitta, T. Nobe, Y. Noguchi, M. Nomachi, I. Nomidis, M. A. Nomura, T. Nooney, M. Nordberg, N. Norjoharuddeen, O. Novgorodova, M. Nozaki, L. Nozka, K. Ntekas, E. Nurse, F. Nuti, K. O’connor, D. C. O’Neil, A. A. O’Rourke, V. O’Shea, F. G. Oakham, H. Oberlack, T. Obermann, J. Ocariz, A. Ochi, I. Ochoa, J. P. Ochoa-Ricoux, S. Oda, S. Odaka, A. Oh, S. H. Oh, C. C. Ohm, H. Ohman, H. Oide, H. Okawa, Y. Okumura, T. Okuyama, A. Olariu, L. F. Oleiro Seabra, S. A. Olivares Pino, D. Oliveira Damazio, A. Olszewski, J. Olszowska, A. Onofre, K. Onogi, P. U. E. Onyisi, H. Oppen, M. J. Oreglia, Y. Oren, D. Orestano, N. Orlando, R. S. Orr, B. Osculati, R. Ospanov, G. Otero y Garzon, H. Otono, M. Ouchrif, F. Ould-Saada, A. Ouraou, K. P. Oussoren, Q. Ouyang, M. Owen, R. E. Owen, V. E. Ozcan, N. Ozturk, K. Pachal, A. Pacheco Pages, L. Pacheco Rodriguez, C. Padilla Aranda, S. Pagan Griso, M. Paganini, F. Paige, G. Palacino, S. Palazzo, S. Palestini, M. Palka, D. Pallin, E. St. Panagiotopoulou, I. Panagoulias, C. E. Pandini, J. G. Panduro Vazquez, P. Pani, S. Panitkin, D. Pantea, L. Paolozzi, Th. D. Papadopoulou, K. Papageorgiou, A. Paramonov, D. Paredes Hernandez, A. J. Parker, M. A. Parker, K. A. Parker, F. Parodi, J. A. Parsons, U. Parzefall, V. R. Pascuzzi, J. M. Pasner, E. Pasqualucci, S. Passaggio, Fr. Pastore, S. Pataraia, J. R. Pater, T. Pauly, B. Pearson, S. Pedraza Lopez, R. Pedro, S. V. Peleganchuk, O. Penc, C. Peng, H. Peng, J. Penwell, B. S. Peralva, M. M. Perego, D. V. Perepelitsa, F. Peri, L. Perini, H. Pernegger, S. Perrella, R. Peschke, V. D. Peshekhonov, K. Peters, R. F. Y. Peters, B. A. Petersen, T. C. Petersen, E. Petit, A. Petridis, C. Petridou, P. Petroff, E. Petrolo, M. Petrov, F. Petrucci, N. E. Pettersson, A. Peyaud, R. Pezoa, F. H. Phillips, P. W. Phillips, G. Piacquadio, E. Pianori, A. Picazio, E. Piccaro, M. A. Pickering, R. Piegaia, J. E. Pilcher, A. D. Pilkington, A. W. J. Pin, M. Pinamonti, J. L. Pinfold, H. Pirumov, M. Pitt, L. Plazak, M.-A. Pleier, V. Pleskot, E. Plotnikova, D. Pluth, P. Podberezko, R. Poettgen, R. Poggi, L. Poggioli, D. Pohl, G. Polesello, A. Poley, A. Policicchio, R. Polifka, A. Polini, C. S. Pollard, V. Polychronakos, K. Pommès, D. Ponomarenko, L. Pontecorvo, G. A. Popeneciu, S. Pospisil, K. Potamianos, I. N. Potrap, C. J. Potter, T. Poulsen, J. Poveda, M. E. Pozo Astigarraga, P. Pralavorio, A. Pranko, S. Prell, D. Price, M. Primavera, S. Prince, N. Proklova, K. Prokofiev, F. Prokoshin, S. Protopopescu, J. Proudfoot, M. Przybycien, A. Puri, P. Puzo, J. Qian, G. Qin, Y. Qin, A. Quadt, M. Queitsch-Maitland, D. Quilty, S. Raddum, V. Radeka, V. Radescu, S. K. Radhakrishnan, P. Radloff, P. Rados, F. Ragusa, G. Rahal, J. A. Raine, S. Rajagopalan, C. Rangel-Smith, T. Rashid, S. Raspopov, M. G. Ratti, D. M. Rauch, F. Rauscher, S. Rave, I. Ravinovich, J. H. Rawling, M. Raymond, A. L. Read, N. P. Readioff, M. Reale, D. M. Rebuzzi, A. Redelbach, G. Redlinger, R. Reece, R. G. Reed, K. Reeves, L. Rehnisch, J. Reichert, A. Reiss, C. Rembser, H. Ren, M. Rescigno, S. Resconi, E. D. Resseguie, S. Rettie, E. Reynolds, O. L. Rezanova, P. Reznicek, R. Rezvani, R. Richter, S. Richter, E. Richter-Was, O. Ricken, M. Ridel, P. Rieck, C. J. Riegel, J. Rieger, O. Rifki, M. Rijssenbeek, A. Rimoldi, M. Rimoldi, L. Rinaldi, G. Ripellino, B. Ristić, E. Ritsch, I. Riu, F. Rizatdinova, E. Rizvi, C. Rizzi, R. T. Roberts, S. H. Robertson, A. Robichaud-Veronneau, D. Robinson, J. E. M. Robinson, A. Robson, E. Rocco, C. Roda, Y. Rodina, S. Rodriguez Bosca, A. Rodriguez Perez, D. Rodriguez Rodriguez, S. Roe, C. S. Rogan, O. Røhne, J. Roloff, A. Romaniouk, M. Romano, S. M. Romano Saez, E. Romero Adam, N. Rompotis, M. Ronzani, L. Roos, S. Rosati, K. Rosbach, P. Rose, N.-A. Rosien, E. Rossi, L. P. Rossi, J. H. N. Rosten, R. Rosten, M. Rotaru, J. Rothberg, D. Rousseau, A. Rozanov, Y. Rozen, X. Ruan, F. Rubbo, F. Rühr, A. Ruiz-Martinez, Z. Rurikova, N. A. Rusakovich, H. L. Russell, J. P. Rutherfoord, N. Ruthmann, Y. F. Ryabov, M. Rybar, G. Rybkin, S. Ryu, A. Ryzhov, G. F. Rzehorz, A. F. Saavedra, G. Sabato, S. Sacerdoti, H.F-W. Sadrozinski, R. Sadykov, F. Safai Tehrani, P. Saha, M. Sahinsoy, M. Saimpert, M. Saito, T. Saito, H. Sakamoto, Y. Sakurai, G. Salamanna, J. E. Salazar Loyola, D. Salek, P. H. Sales De Bruin, D. Salihagic, A. Salnikov, J. Salt, D. Salvatore, F. Salvatore, A. Salvucci, A. Salzburger, D. Sammel, D. Sampsonidis, D. Sampsonidou, J. Sánchez, V. Sanchez Martinez, A. Sanchez Pineda, H. Sandaker, R. L. Sandbach, C. O. Sander, M. Sandhoff, C. Sandoval, D. P. C. Sankey, M. Sannino, Y. Sano, A. Sansoni, C. Santoni, H. Santos, I. Santoyo Castillo, A. Sapronov, J. G. Saraiva, B. Sarrazin, O. Sasaki, K. Sato, E. Sauvan, G. Savage, P. Savard, N. Savic, C. Sawyer, L. Sawyer, J. Saxon, C. Sbarra, A. Sbrizzi, T. Scanlon, D. A. Scannicchio, M. Scarcella, J. Schaarschmidt, P. Schacht, B. M. Schachtner, D. Schaefer, L. Schaefer, R. Schaefer, J. Schaeffer, S. Schaepe, S. Schaetzel, U. Schäfer, A. C. Schaffer, D. Schaile, R. D. Schamberger, V. A. Schegelsky, D. Scheirich, M. Schernau, C. Schiavi, S. Schier, L. K. Schildgen, C. Schillo, M. Schioppa, S. Schlenker, K. R. Schmidt-Sommerfeld, K. Schmieden, C. Schmitt, S. Schmitt, S. Schmitz, U. Schnoor, L. Schoeffel, A. Schoening, B. D. Schoenrock, E. Schopf, M. Schott, J. F. P. Schouwenberg, J. Schovancova, S. Schramm, N. Schuh, A. Schulte, M. J. Schultens, H.-C. Schultz-Coulon, H. Schulz, M. Schumacher, B. A. Schumm, Ph. Schune, A. Schwartzman, T. A. Schwarz, H. Schweiger, Ph. Schwemling, R. Schwienhorst, J. Schwindling, A. Sciandra, G. Sciolla, M. Scornajenghi, F. Scuri, F. Scutti, J. Searcy, P. Seema, S. C. Seidel, A. Seiden, J. M. Seixas, G. Sekhniaidze, K. Sekhon, S. J. Sekula, N. Semprini-Cesari, S. Senkin, C. Serfon, L. Serin, L. Serkin, M. Sessa, R. Seuster, H. Severini, T. Sfiligoj, F. Sforza, A. Sfyrla, E. Shabalina, N. W. Shaikh, L. Y. Shan, R. Shang, J. T. Shank, M. Shapiro, P. B. Shatalov, K. Shaw, S. M. Shaw, A. Shcherbakova, C. Y. Shehu, Y. Shen, N. Sherafati, P. Sherwood, L. Shi, S. Shimizu, C. O. Shimmin, M. Shimojima, I. P. J. Shipsey, S. Shirabe, M. Shiyakova, J. Shlomi, A. Shmeleva, D. Shoaleh Saadi, M. J. Shochet, S. Shojaii, D. R. Shope, S. Shrestha, E. Shulga, M. A. Shupe, P. Sicho, A. M. Sickles, P. E. Sidebo, E. Sideras Haddad, O. Sidiropoulou, A. Sidoti, F. Siegert, Dj. Sijacki, J. Silva, S. B. Silverstein, V. Simak, Lj. Simic, S. Simion, E. Simioni, B. Simmons, M. Simon, P. Sinervo, N. B. Sinev, M. Sioli, G. Siragusa, I. Siral, S. Yu. Sivoklokov, J. Sjölin, M. B. Skinner, P. Skubic, M. Slater, T. Slavicek, M. Slawinska, K. Sliwa, R. Slovak, V. Smakhtin, B. H. Smart, J. Smiesko, N. Smirnov, S. Yu. Smirnov, Y. Smirnov, L. N. Smirnova, O. Smirnova, J. W. Smith, M. N. K. Smith, R. W. Smith, M. Smizanska, K. Smolek, A. A. Snesarev, I. M. Snyder, S. Snyder, R. Sobie, F. Socher, A. Soffer, A. Søgaard, D. A. Soh, G. Sokhrannyi, C. A. Solans Sanchez, M. Solar, E. Yu. Soldatov, U. Soldevila, A. A. Solodkov, A. Soloshenko, O. V. Solovyanov, V. Solovyev, P. Sommer, H. Son, A. Sopczak, D. Sosa, C. L. Sotiropoulou, R. Soualah, A. M. Soukharev, D. South, B. C. Sowden, S. Spagnolo, M. Spalla, M. Spangenberg, F. Spanò, D. Sperlich, F. Spettel, T. M. Spieker, R. Spighi, G. Spigo, L. A. Spiller, M. Spousta, R. D. St. Denis, A. Stabile, R. Stamen, S. Stamm, E. Stanecka, R. W. Stanek, C. Stanescu, M. M. Stanitzki, B. S. Stapf, S. Stapnes, E. A. Starchenko, G. H. Stark, J. Stark, S. H Stark, P. Staroba, P. Starovoitov, S. Stärz, R. Staszewski, P. Steinberg, B. Stelzer, H. J. Stelzer, O. Stelzer-Chilton, H. Stenzel, G. A. Stewart, M. C. Stockton, M. Stoebe, G. Stoicea, P. Stolte, S. Stonjek, A. R. Stradling, A. Straessner, M. E. Stramaglia, J. Strandberg, S. Strandberg, M. Strauss, P. Strizenec, R. Ströhmer, D. M. Strom, R. Stroynowski, A. Strubig, S. A. Stucci, B. Stugu, N. A. Styles, D. Su, J. Su, S. Suchek, Y. Sugaya, M. Suk, V. V. Sulin, DMS Sultan, S. Sultansoy, T. Sumida, S. Sun, X. Sun, K. Suruliz, C. J. E. Suster, M. R. Sutton, S. Suzuki, M. Svatos, M. Swiatlowski, S. P. Swift, I. Sykora, T. Sykora, D. Ta, K. Tackmann, J. Taenzer, A. Taffard, R. Tafirout, E. Tahirovic, N. Taiblum, H. Takai, R. Takashima, E. H. Takasugi, T. Takeshita, Y. Takubo, M. Talby, A. A. Talyshev, J. Tanaka, M. Tanaka, R. Tanaka, S. Tanaka, R. Tanioka, B. B. Tannenwald, S. Tapia Araya, S. Tapprogge, S. Tarem, G. F. Tartarelli, P. Tas, M. Tasevsky, T. Tashiro, E. Tassi, A. Tavares Delgado, Y. Tayalati, A. C. Taylor, G. N. Taylor, P. T. E. Taylor, W. Taylor, P. Teixeira-Dias, D. Temple, H. Ten Kate, P. K. Teng, J. J. Teoh, F. Tepel, S. Terada, K. Terashi, J. Terron, S. Terzo, M. Testa, R. J. Teuscher, T. Theveneaux-Pelzer, F. Thiele, J. P. Thomas, J. Thomas-Wilsker, P. D. Thompson, A. S. Thompson, L. A. Thomsen, E. Thomson, M. J. Tibbetts, R. E. Ticse Torres, V. O. Tikhomirov, Yu. A. Tikhonov, S. Timoshenko, P. Tipton, S. Tisserant, K. Todome, S. Todorova-Nova, S. Todt, J. Tojo, S. Tokár, K. Tokushuku, E. Tolley, L. Tomlinson, M. Tomoto, L. Tompkins, K. Toms, B. Tong, P. Tornambe, E. Torrence, H. Torres, E. Torró Pastor, J. Toth, F. Touchard, D. R. Tovey, C. J. Treado, T. Trefzger, F. Tresoldi, A. Tricoli, I. M. Trigger, S. Trincaz-Duvoid, M. F. Tripiana, W. Trischuk, B. Trocmé, A. Trofymov, C. Troncon, M. Trottier-McDonald, M. Trovatelli, L. Truong, M. Trzebinski, A. Trzupek, K. W. Tsang, J. C-L. Tseng, P. V. Tsiareshka, G. Tsipolitis, N. Tsirintanis, S. Tsiskaridze, V. Tsiskaridze, E. G. Tskhadadze, K. M. Tsui, I. I. Tsukerman, V. Tsulaia, S. Tsuno, D. Tsybychev, Y. Tu, A. Tudorache, V. Tudorache, T. T. Tulbure, A. N. Tuna, S. A. Tupputi, S. Turchikhin, D. Turgeman, I. Turk Cakir, R. Turra, P. M. Tuts, G. Ucchielli, I. Ueda, M. Ughetto, F. Ukegawa, G. Unal, A. Undrus, G. Unel, F. C. Ungaro, Y. Unno, C. Unverdorben, J. Urban, P. Urquijo, P. Urrejola, G. Usai, J. Usui, L. Vacavant, V. Vacek, B. Vachon, K. O. H. Vadla, A. Vaidya, C. Valderanis, E. Valdes Santurio, M. Valente, S. Valentinetti, A. Valero, L. Valéry, S. Valkar, A. Vallier, J. A. Valls Ferrer, W. Van Den Wollenberg, H. van der Graaf, P. van Gemmeren, J. Van Nieuwkoop, I. van Vulpen, M. C. van Woerden, M. Vanadia, W. Vandelli, A. Vaniachine, P. Vankov, G. Vardanyan, R. Vari, E. W. Varnes, C. Varni, T. Varol, D. Varouchas, A. Vartapetian, K. E. Varvell, J. G. Vasquez, G. A. Vasquez, F. Vazeille, T. Vazquez Schroeder, J. Veatch, V. Veeraraghavan, L. M. Veloce, F. Veloso, S. Veneziano, A. Ventura, M. Venturi, N. Venturi, A. Venturini, V. Vercesi, M. Verducci, W. Verkerke, A. T. Vermeulen, J. C. Vermeulen, M. C. Vetterli, N. Viaux Maira, O. Viazlo, I. Vichou, T. Vickey, O. E. Vickey Boeriu, G. H. A. Viehhauser, S. Viel, L. Vigani, M. Villa, M. Villaplana Perez, E. Vilucchi, M. G. Vincter, V. B. Vinogradov, A. Vishwakarma, C. Vittori, I. Vivarelli, S. Vlachos, M. Vogel, P. Vokac, G. Volpi, H. von der Schmitt, E. von Toerne, V. Vorobel, K. Vorobev, M. Vos, R. Voss, J. H. Vossebeld, N. Vranjes, M. Vranjes Milosavljevic, V. Vrba, M. Vreeswijk, R. Vuillermet, I. Vukotic, P. Wagner, W. Wagner, J. Wagner-Kuhr, H. Wahlberg, S. Wahrmund, J. Wakabayashi, J. Walder, R. Walker, W. Walkowiak, V. Wallangen, C. Wang, C. Wang, F. Wang, H. Wang, H. Wang, J. Wang, J. Wang, Q. Wang, R. Wang, S. M. Wang, T. Wang, W. Wang, W. Wang, Z. Wang, C. Wanotayaroj, A. Warburton, C. P. Ward, D. R. Wardrope, A. Washbrook, P. M. Watkins, A. T. Watson, M. F. Watson, G. Watts, S. Watts, B. M. Waugh, A. F. Webb, S. Webb, M. S. Weber, S. W. Weber, S. A. Weber, J. S. Webster, A. R. Weidberg, B. Weinert, J. Weingarten, M. Weirich, C. Weiser, H. Weits, P. S. Wells, T. Wenaus, T. Wengler, S. Wenig, N. Wermes, M. D. Werner, P. Werner, M. Wessels, T. D. Weston, K. Whalen, N. L. Whallon, A. M. Wharton, A. S. White, A. White, M. J. White, R. White, D. Whiteson, B. W. Whitmore, F. J. Wickens, W. Wiedenmann, M. Wielers, C. Wiglesworth, L. A. M. Wiik-Fuchs, A. Wildauer, F. Wilk, H. G. Wilkens, H. H. Williams, S. Williams, C. Willis, S. Willocq, J. A. Wilson, I. Wingerter-Seez, E. Winkels, F. Winklmeier, O. J. Winston, B. T. Winter, M. Wittgen, M. Wobisch, T. M. H. Wolf, R. Wolff, M. W. Wolter, H. Wolters, V. W. S. Wong, S. D. Worm, B. K. Wosiek, J. Wotschack, K. W. Wozniak, M. Wu, S. L. Wu, X. Wu, Y. Wu, T. R. Wyatt, B. M. Wynne, S. Xella, Z. Xi, L. Xia, D. Xu, L. Xu, T. Xu, B. Yabsley, S. Yacoob, D. Yamaguchi, Y. Yamaguchi, A. Yamamoto, S. Yamamoto, T. Yamanaka, M. Yamatani, K. Yamauchi, Y. Yamazaki, Z. Yan, H. Yang, H. Yang, Y. Yang, Z. Yang, W-M. Yao, Y. C. Yap, Y. Yasu, E. Yatsenko, K. H. Yau Wong, J. Ye, S. Ye, I. Yeletskikh, E. Yigitbasi, E. Yildirim, K. Yorita, K. Yoshihara, C. Young, C. J. S. Young, J. Yu, J. Yu, S. P. Y. Yuen, I. Yusuff, B. Zabinski, G. Zacharis, R. Zaidan, A. M. Zaitsev, N. Zakharchuk, J. Zalieckas, A. Zaman, S. Zambito, D. Zanzi, C. Zeitnitz, G. Zemaityte, A. Zemla, J. C. Zeng, Q. Zeng, O. Zenin, T. Ženiš, D. Zerwas, D. Zhang, F. Zhang, G. Zhang, H. Zhang, J. Zhang, L. Zhang, L. Zhang, M. Zhang, P. Zhang, R. Zhang, R. Zhang, X. Zhang, Y. Zhang, Z. Zhang, X. Zhao, Y. Zhao, Z. Zhao, A. Zhemchugov, B. Zhou, C. Zhou, L. Zhou, M. Zhou, M. Zhou, N. Zhou, C. G. Zhu, H. Zhu, J. Zhu, Y. Zhu, X. Zhuang, K. Zhukov, A. Zibell, D. Zieminska, N. I. Zimine, C. Zimmermann, S. Zimmermann, Z. Zinonos, M. Zinser, M. Ziolkowski, L. Živković, G. Zobernig, A. Zoccoli, R. Zou, M. zur Nedden, L. Zwalinski, ATLAS Collaboration

**Affiliations:** 1 Department of Physics, University of Adelaide, Adelaide, Australia; 2 Physics Department, SUNY Albany, Albany, NY USA; 3 Department of Physics, University of Alberta, Edmonton, AB Canada; 4 Department of Physics, Ankara University, Ankara, Turkey; 5 Istanbul Aydin University, Istanbul, Turkey; 6 Division of Physics, TOBB University of Economics and Technology, Ankara, Turkey; 7 LAPP, CNRS/IN2P3 and Université Savoie Mont Blanc, Annecy-le-Vieux, France; 8 High Energy Physics Division, Argonne National Laboratory, Argonne, IL USA; 9 Department of Physics, University of Arizona, Tucson, AZ USA; 10 Department of Physics, The University of Texas at Arlington, Arlington, TX USA; 11 Physics Department, National and Kapodistrian University of Athens, Athens, Greece; 12 Physics Department, National Technical University of Athens, Zografou, Greece; 13 Department of Physics, The University of Texas at Austin, Austin, TX USA; 14 Institute of Physics, Azerbaijan Academy of Sciences, Baku, Azerbaijan; 15 Institut de Física d’Altes Energies (IFAE), The Barcelona Institute of Science and Technology, Barcelona, Spain; 16 Institute of Physics, University of Belgrade, Belgrade, Serbia; 17 Department for Physics and Technology, University of Bergen, Bergen, Norway; 18 Physics Division, Lawrence Berkeley National Laboratory and University of California, Berkeley, CA USA; 19 Department of Physics, Humboldt University, Berlin, Germany; 20 Albert Einstein Center for Fundamental Physics and Laboratory for High Energy Physics, University of Bern, Bern, Switzerland; 21 School of Physics and Astronomy, University of Birmingham, Birmingham, UK; 22 Department of Physics, Bogazici University, Istanbul, Turkey; 23 Department of Physics Engineering, Gaziantep University, Gaziantep, Turkey; 24 Faculty of Engineering and Natural Sciences, Istanbul Bilgi University, Istanbul, Turkey; 25 Faculty of Engineering and Natural Sciences, Bahcesehir University, Istanbul, Turkey; 26 Centro de Investigaciones, Universidad Antonio Narino, Bogotá, Colombia; 27 INFN Sezione di Bologna, Bologna, Italy; 28 Dipartimento di Fisica e Astronomia, Università di Bologna, Bologna, Italy; 29 Physikalisches Institut, University of Bonn, Bonn, Germany; 30 Department of Physics, Boston University, Boston, MA USA; 31 Department of Physics, Brandeis University, Waltham, MA USA; 32 Universidade Federal do Rio De Janeiro COPPE/EE/IF, Rio de Janeiro, Brazil; 33 Electrical Circuits Department, Federal University of Juiz de Fora (UFJF), Juiz de Fora, Brazil; 34 Federal University of Sao Joao del Rei (UFSJ), Sao Joao del Rei, Brazil; 35 Instituto de Fisica, Universidade de Sao Paulo, Sao Paulo, Brazil; 36 Physics Department, Brookhaven National Laboratory, Upton, NY USA; 37 Transilvania University of Brasov, Brasov, Romania; 38 Horia Hulubei National Institute of Physics and Nuclear Engineering, Bucharest, Romania; 39 Department of Physics, Alexandru Ioan Cuza University of Iasi, Iasi, Romania; 40 Physics Department, National Institute for Research and Development of Isotopic and Molecular Technologies, Cluj Napoca, Romania; 41 University Politehnica Bucharest, Bucharest, Romania; 42 West University in Timisoara, Timisoara, Romania; 43 Departamento de Física, Universidad de Buenos Aires, Buenos Aires, Argentina; 44 Cavendish Laboratory, University of Cambridge, Cambridge, UK; 45 Department of Physics, Carleton University, Ottawa, ON Canada; 46 CERN, Geneva, Switzerland; 47 Enrico Fermi Institute, University of Chicago, Chicago, IL USA; 48 Departamento de Física, Pontificia Universidad Católica de Chile, Santiago, Chile; 49 Departamento de Física, Universidad Técnica Federico Santa María, Valparaiso, Chile; 50 Institute of High Energy Physics, Chinese Academy of Sciences, Beijing, China; 51 Department of Physics, Nanjing University, Nanjing, Jiangsu China; 52 Physics Department, Tsinghua University, Beijing, 100084 China; 53 Department of Modern Physics and State Key Laboratory of Particle Detection and Electronics, University of Science and Technology of China, Hefei, Anhui China; 54 School of Physics, Shandong University, Shandong, China; 55 Department of Physics and Astronomy, Key Laboratory for Particle Physics, Astrophysics and Cosmology, Ministry of Education, Shanghai Key Laboratory for Particle Physics and Cosmology, Shanghai Jiao Tong University (also at PKU-CHEP), Shanghai, China; 56 Université Clermont Auvergne, CNRS/IN2P3, LPC, Clermont-Ferrand, France; 57 Nevis Laboratory, Columbia University, Irvington, NY USA; 58 Niels Bohr Institute, University of Copenhagen, Copenhagen, Denmark; 59 INFN Gruppo Collegato di Cosenza, Laboratori Nazionali di Frascati, Frascati, Italy; 60 Dipartimento di Fisica, Università della Calabria, Rende, Italy; 61 Faculty of Physics and Applied Computer Science, AGH University of Science and Technology, Kraków, Poland; 62 Marian Smoluchowski Institute of Physics, Jagiellonian University, Kraków, Poland; 63 Institute of Nuclear Physics, Polish Academy of Sciences, Kraków, Poland; 64 Physics Department, Southern Methodist University, Dallas, TX USA; 65 Physics Department, University of Texas at Dallas, Richardson, TX USA; 66 DESY, Hamburg and Zeuthen, Germany; 67 Lehrstuhl für Experimentelle Physik IV, Technische Universität Dortmund, Dortmund, Germany; 68 Institut für Kern- und Teilchenphysik, Technische Universität Dresden, Dresden, Germany; 69 Department of Physics, Duke University, Durham, NC USA; 70 SUPA-School of Physics and Astronomy, University of Edinburgh, Edinburgh, UK; 71 INFN e Laboratori Nazionali di Frascati, Frascati, Italy; 72 Fakultät für Mathematik und Physik, Albert-Ludwigs-Universität, Freiburg, Germany; 73 Departement de Physique Nucleaire et Corpusculaire, Université de Genève, Geneva, Switzerland; 74 INFN Sezione di Genova, Genoa, Italy; 75 Dipartimento di Fisica, Università di Genova, Genoa, Italy; 76 E. Andronikashvili Institute of Physics, Iv. Javakhishvili Tbilisi State University, Tbilisi, Georgia; 77 High Energy Physics Institute, Tbilisi State University, Tbilisi, Georgia; 78 II Physikalisches Institut, Justus-Liebig-Universität Giessen, Giessen, Germany; 79 SUPA-School of Physics and Astronomy, University of Glasgow, Glasgow, UK; 80 II Physikalisches Institut, Georg-August-Universität, Göttingen, Germany; 81 Laboratoire de Physique Subatomique et de Cosmologie, Université Grenoble-Alpes, CNRS/IN2P3, Grenoble, France; 82 Laboratory for Particle Physics and Cosmology, Harvard University, Cambridge, MA USA; 83 Kirchhoff-Institut für Physik, Ruprecht-Karls-Universität Heidelberg, Heidelberg, Germany; 84 Physikalisches Institut, Ruprecht-Karls-Universität Heidelberg, Heidelberg, Germany; 85 Faculty of Applied Information Science, Hiroshima Institute of Technology, Hiroshima, Japan; 86 Department of Physics, The Chinese University of Hong Kong, Shatin, N.T. Hong Kong; 87 Department of Physics, The University of Hong Kong, Hong Kong, China; 88 Department of Physics and Institute for Advanced Study, The Hong Kong University of Science and Technology, Clear Water Bay, Kowloon, Hong Kong, China; 89 Department of Physics, National Tsing Hua University, Taiwan, Taiwan; 90 Department of Physics, Indiana University, Bloomington, IN USA; 91 Institut für Astro- und Teilchenphysik, Leopold-Franzens-Universität, Innsbruck, Austria; 92 University of Iowa, Iowa City, IA USA; 93 Department of Physics and Astronomy, Iowa State University, Ames, IA USA; 94 Joint Institute for Nuclear Research, JINR Dubna, Dubna, Russia; 95 KEK, High Energy Accelerator Research Organization, Tsukuba, Japan; 96 Graduate School of Science, Kobe University, Kobe, Japan; 97 Faculty of Science, Kyoto University, Kyoto, Japan; 98 Kyoto University of Education, Kyoto, Japan; 99 Research Center for Advanced Particle Physics and Department of Physics, Kyushu University, Fukuoka, Japan; 100 Instituto de Física La Plata, Universidad Nacional de La Plata and CONICET, La Plata, Argentina; 101 Physics Department, Lancaster University, Lancaster, UK; 102 INFN Sezione di Lecce, Lecce, Italy; 103 Dipartimento di Matematica e Fisica, Università del Salento, Lecce, Italy; 104 Oliver Lodge Laboratory, University of Liverpool, Liverpool, UK; 105 Department of Experimental Particle Physics, Jožef Stefan Institute and Department of Physics, University of Ljubljana, Ljubljana, Slovenia; 106 School of Physics and Astronomy, Queen Mary University of London, London, UK; 107 Department of Physics, Royal Holloway University of London, Surrey, UK; 108 Department of Physics and Astronomy, University College London, London, UK; 109 Louisiana Tech University, Ruston, LA USA; 110 Laboratoire de Physique Nucléaire et de Hautes Energies, UPMC and Université Paris-Diderot and CNRS/IN2P3, Paris, France; 111 Fysiska institutionen, Lunds universitet, Lund, Sweden; 112 Departamento de Fisica Teorica C-15, Universidad Autonoma de Madrid, Madrid, Spain; 113 Institut für Physik, Universität Mainz, Mainz, Germany; 114 School of Physics and Astronomy, University of Manchester, Manchester, UK; 115 CPPM, Aix-Marseille Université and CNRS/IN2P3, Marseille, France; 116 Department of Physics, University of Massachusetts, Amherst, MA USA; 117 Department of Physics, McGill University, Montreal, QC Canada; 118 School of Physics, University of Melbourne, Victoria, Australia; 119 Department of Physics, The University of Michigan, Ann Arbor, MI USA; 120 Department of Physics and Astronomy, Michigan State University, East Lansing, MI USA; 121 INFN Sezione di Milano, Milan, Italy; 122 Dipartimento di Fisica, Università di Milano, Milan, Italy; 123 B.I. Stepanov Institute of Physics, National Academy of Sciences of Belarus, Minsk, Republic of Belarus; 124 Research Institute for Nuclear Problems of Byelorussian State University, Minsk, Republic of Belarus; 125 Group of Particle Physics, University of Montreal, Montreal, QC Canada; 126 P.N. Lebedev Physical Institute of the Russian Academy of Sciences, Moscow, Russia; 127 Institute for Theoretical and Experimental Physics (ITEP), Moscow, Russia; 128 National Research Nuclear University MEPhI, Moscow, Russia; 129 D.V. Skobeltsyn Institute of Nuclear Physics, M.V. Lomonosov Moscow State University, Moscow, Russia; 130 Fakultät für Physik, Ludwig-Maximilians-Universität München, Munich, Germany; 131 Max-Planck-Institut für Physik (Werner-Heisenberg-Institut), Munich, Germany; 132 Nagasaki Institute of Applied Science, Nagasaki, Japan; 133 Graduate School of Science and Kobayashi-Maskawa Institute, Nagoya University, Nagoya, Japan; 134 INFN Sezione di Napoli, Naples, Italy; 135 Dipartimento di Fisica, Università di Napoli, Naples, Italy; 136 Department of Physics and Astronomy, University of New Mexico, Albuquerque, NM USA; 137 Institute for Mathematics, Astrophysics and Particle Physics, Radboud University Nijmegen/Nikhef, Nijmegen, The Netherlands; 138 Nikhef National Institute for Subatomic Physics and University of Amsterdam, Amsterdam, The Netherlands; 139 Department of Physics, Northern Illinois University, DeKalb, IL USA; 140 Budker Institute of Nuclear Physics, SB RAS, Novosibirsk, Russia; 141 Department of Physics, New York University, New York, NY USA; 142 Ohio State University, Columbus, OH USA; 143 Faculty of Science, Okayama University, Okayama, Japan; 144 Homer L. Dodge Department of Physics and Astronomy, University of Oklahoma, Norman, OK USA; 145 Department of Physics, Oklahoma State University, Stillwater, OK USA; 146 Palacký University, RCPTM, Olomouc, Czech Republic; 147 Center for High Energy Physics, University of Oregon, Eugene, OR USA; 148 LAL, Univ. Paris-Sud, CNRS/IN2P3, Université Paris-Saclay, Orsay, France; 149 Graduate School of Science, Osaka University, Osaka, Japan; 150 Department of Physics, University of Oslo, Oslo, Norway; 151 Department of Physics, Oxford University, Oxford, UK; 152 INFN Sezione di Pavia, Pavia, Italy; 153 Dipartimento di Fisica, Università di Pavia, Pavia, Italy; 154 Department of Physics, University of Pennsylvania, Philadelphia, PA USA; 155 National Research Centre “Kurchatov Institute” B.P. Konstantinov Petersburg Nuclear Physics Institute, St. Petersburg, Russia; 156 INFN Sezione di Pisa, Pisa, Italy; 157 Dipartimento di Fisica E. Fermi, Università di Pisa, Pisa, Italy; 158 Department of Physics and Astronomy, University of Pittsburgh, Pittsburgh, PA USA; 159 Laboratório de Instrumentação e Física Experimental de Partículas-LIP, Lisbon, Portugal; 160 Faculdade de Ciências, Universidade de Lisboa, Lisbon, Portugal; 161 Department of Physics, University of Coimbra, Coimbra, Portugal; 162 Centro de Física Nuclear da Universidade de Lisboa, Lisbon, Portugal; 163 Departamento de Fisica, Universidade do Minho, Braga, Portugal; 164 Departamento de Fisica Teorica y del Cosmos, Universidad de Granada, Granada, Spain; 165 Dep Fisica and CEFITEC of Faculdade de Ciencias e Tecnologia, Universidade Nova de Lisboa, Caparica, Portugal; 166 Institute of Physics, Academy of Sciences of the Czech Republic, Prague, Czech Republic; 167 Czech Technical University in Prague, Prague, Czech Republic; 168 Faculty of Mathematics and Physics, Charles University, Prague, Czech Republic; 169 State Research Center Institute for High Energy Physics (Protvino), NRC KI, Protvino, Russia; 170 Particle Physics Department, Rutherford Appleton Laboratory, Didcot, UK; 171 INFN Sezione di Roma, Rome, Italy; 172 Dipartimento di Fisica, Sapienza Università di Roma, Rome, Italy; 173 INFN Sezione di Roma Tor Vergata, Rome, Italy; 174 Dipartimento di Fisica, Università di Roma Tor Vergata, Rome, Italy; 175 INFN Sezione di Roma Tre, Rome, Italy; 176 Dipartimento di Matematica e Fisica, Università Roma Tre, Rome, Italy; 177 Faculté des Sciences Ain Chock, Réseau Universitaire de Physique des Hautes Energies-Université Hassan II, Casablanca, Morocco; 178 Centre National de l’Energie des Sciences Techniques Nucleaires, Rabat, Morocco; 179 Faculté des Sciences Semlalia, Université Cadi Ayyad, LPHEA-Marrakech, Marrakech, Morocco; 180 Faculté des Sciences, Université Mohamed Premier and LPTPM, Oujda, Morocco; 181 Faculté des Sciences, Université Mohammed V, Rabat, Morocco; 182 DSM/IRFU (Institut de Recherches sur les Lois Fondamentales de l’Univers), CEA Saclay (Commissariat à l’Energie Atomique et aux Energies Alternatives), Gif-sur-Yvette, France; 183 Santa Cruz Institute for Particle Physics, University of California Santa Cruz, Santa Cruz, CA USA; 184 Department of Physics, University of Washington, Seattle, WA USA; 185 Department of Physics and Astronomy, University of Sheffield, Sheffield, UK; 186 Department of Physics, Shinshu University, Nagano, Japan; 187 Department Physik, Universität Siegen, Siegen, Germany; 188 Department of Physics, Simon Fraser University, Burnaby, BC Canada; 189 SLAC National Accelerator Laboratory, Stanford, CA USA; 190 Faculty of Mathematics, Physics and Informatics, Comenius University, Bratislava, Slovak Republic; 191 Department of Subnuclear Physics, Institute of Experimental Physics of the Slovak Academy of Sciences, Kosice, Slovak Republic; 192 Department of Physics, University of Cape Town, Cape Town, South Africa; 193 Department of Physics, University of Johannesburg, Johannesburg, South Africa; 194 School of Physics, University of the Witwatersrand, Johannesburg, South Africa; 195 Department of Physics, Stockholm University, Stockholm, Sweden; 196 The Oskar Klein Centre, Stockholm, Sweden; 197 Physics Department, Royal Institute of Technology, Stockholm, Sweden; 198 Departments of Physics and Astronomy and Chemistry, Stony Brook University, Stony Brook, NY USA; 199 Department of Physics and Astronomy, University of Sussex, Brighton, UK; 200 School of Physics, University of Sydney, Sydney, Australia; 201 Institute of Physics, Academia Sinica, Taipei, Taiwan; 202 Department of Physics, Technion: Israel Institute of Technology, Haifa, Israel; 203 Raymond and Beverly Sackler School of Physics and Astronomy, Tel Aviv University, Tel Aviv, Israel; 204 Department of Physics, Aristotle University of Thessaloniki, Thessaloniki, Greece; 205 International Center for Elementary Particle Physics and Department of Physics, The University of Tokyo, Tokyo, Japan; 206 Graduate School of Science and Technology, Tokyo Metropolitan University, Tokyo, Japan; 207 Department of Physics, Tokyo Institute of Technology, Tokyo, Japan; 208 Tomsk State University, Tomsk, Russia; 209 Department of Physics, University of Toronto, Toronto, ON Canada; 210 INFN-TIFPA, Trento, Italy; 211 University of Trento, Trento, Italy; 212 TRIUMF, Vancouver, BC Canada; 213 Department of Physics and Astronomy, York University, Toronto, ON Canada; 214 Faculty of Pure and Applied Sciences, and Center for Integrated Research in Fundamental Science and Engineering, University of Tsukuba, Tsukuba, Japan; 215 Department of Physics and Astronomy, Tufts University, Medford, MA USA; 216 Department of Physics and Astronomy, University of California Irvine, Irvine, CA USA; 217 INFN Gruppo Collegato di Udine, Sezione di Trieste, Udine, Italy; 218 ICTP, Trieste, Italy; 219 Dipartimento di Chimica, Fisica e Ambiente, Università di Udine, Udine, Italy; 220 Department of Physics and Astronomy, University of Uppsala, Uppsala, Sweden; 221 Department of Physics, University of Illinois, Urbana, IL USA; 222 Instituto de Fisica Corpuscular (IFIC), Centro Mixto Universidad de Valencia-CSIC, Valencia, Spain; 223 Department of Physics, University of British Columbia, Vancouver, BC Canada; 224 Department of Physics and Astronomy, University of Victoria, Victoria, BC Canada; 225 Department of Physics, University of Warwick, Coventry, UK; 226 Waseda University, Tokyo, Japan; 227 Department of Particle Physics, The Weizmann Institute of Science, Rehovot, Israel; 228 Department of Physics, University of Wisconsin, Madison, WI USA; 229 Fakultät für Physik und Astronomie, Julius-Maximilians-Universität, Würzburg, Germany; 230 Fakultät für Mathematik und Naturwissenschaften, Fachgruppe Physik, Bergische Universität Wuppertal, Wuppertal, Germany; 231 Department of Physics, Yale University, New Haven, CT USA; 232 Yerevan Physics Institute, Yerevan, Armenia; 233 Centre de Calcul de l’Institut National de Physique Nucléaire et de Physique des Particules (IN2P3), Villeurbanne, France; 234 Academia Sinica Grid Computing, Institute of Physics, Academia Sinica, Taipei, Taiwan; 235 CERN, 1211 Geneva 23, Switzerland

## Abstract

This paper presents a study of the production of *WW* or *WZ* boson pairs, with one *W* boson decaying to $e\nu$ or $\mu \nu$ and one *W* or *Z* boson decaying hadronically. The analysis uses $20.2~\text{ fb }^{-1}$ of $\sqrt{s} =8~\text {TeV}$ *pp* collision data, collected by the ATLAS detector at the Large Hadron Collider. Cross-sections for *WW* / *WZ* production are measured in high-$p_{\mathrm {T}}$ fiducial regions defined close to the experimental event selection. The cross-section is measured for the case where the hadronically decaying boson is reconstructed as two resolved jets, and the case where it is reconstructed as a single jet. The transverse momentum distribution of the hadronically decaying boson is used to search for new physics. Observations are consistent with the Standard Model predictions, and 95% confidence intervals are calculated for parameters describing anomalous triple gauge-boson couplings.

## Introduction

Measurements of the production of two massive vector gauge bosons (hereafter, “diboson” production) represent an important test of the Standard Model (SM) of particle physics. Diboson measurements are powerful probes of the electroweak theory of the SM, in particular the structure of the triple gauge-boson couplings (TGCs) [1, 2]. In addition, precise diboson measurements are a valuable test of higher-order calculations in quantum chromodynamics (QCD).

Measurements of *WW* and *WZ* production in the leptonic channels $\ell \nu \ell \nu$ and $\ell \nu \ell \ell$ ($\ell =e,\mu$) have been performed by the ATLAS and CMS collaborations in *pp* collisions at $\sqrt{s} =8~\text {TeV}$ and $\sqrt{s} =13~\text {TeV}$ [3–9], and by the Tevatron experiments in $p\bar{p}$ collisions [10–13]. Measurements in the semileptonic channel $WV \rightarrow \ell \nu q q^{\prime }$ ($V=W,Z$) have been performed by ATLAS [14] and CMS [15] at $\sqrt{s} =7~\text {TeV}$, and by the Tevatron experiments in $p\bar{p}$ collisions [16, 17]. The semileptonic channel offers features complementary to the leptonic channels. On the one hand, the presence of jets and the large background from $W+\mathrm {jets}$ and $t\bar{t}$ production limit the experimental precision. On the other hand, the semileptonic channel has an approximately six times higher branching fraction than the fully leptonic channels. Also, for *WW*, the original diboson kinematics can be better reconstructed in an $\ell \nu q q^{\prime }$ final state than in an $\ell \nu \ell \nu$ final state, since the latter has two invisible particles, rather than only one in $\ell \nu q q^{\prime }$. Both of these advantages are particularly beneficial for searching for beyond-the-Standard-Model (BSM) enhancements of diboson production due to heavy new particles, which could modify the diboson spectrum at high transverse momentum ($p_{\mathrm {T}}$) of the bosons [18].

It is possible to reconstruct the $V \rightarrow q q^{\prime }$ decay as two small-radius jets (“small-*R*” jets, denoted by $\mathrm {j}$) or as a single large-radius jet (“large-*R*” jet, denoted by $\mathrm {J}$). Reconstructing the $V \rightarrow q q^{\prime }$ decay as a large-*R* jet enables an increased reconstruction efficiency at high $p_{\mathrm {T}}(V)$, thus improving the sensitivity to BSM signals. In addition, by applying grooming [19] techniques such as trimming [20] to the large-*R* jets, it is possible to better distinguish events containing $V \rightarrow q q^{\prime }$ decays from background events [21].

In this paper, measurements of $WV \rightarrow \ell \nu q q^{\prime }$ fiducial cross-sections are presented in phase spaces containing a $V \rightarrow q q^{\prime }$ candidate with high $p_{\mathrm {T}}$. Two fiducial cross-sections are measured, in phase spaces chosen to closely match the two experimental selections used in this paper. The first event selection, denoted $WV \rightarrow \ell \nu \mathrm {j}\mathrm {j}$, reconstructs the $V \rightarrow q q^{\prime }$ decay as two small-*R* jets, while the second one, denoted $WV \rightarrow \ell \nu \mathrm {J}$, reconstructs the $V \rightarrow q q^{\prime }$ as a single large-*R* jet. Previous cross-section measurements of $WV \rightarrow \ell \nu q q^{\prime }$ have not exploited large-*R* jets.

A search for anomalous triple gauge-boson couplings (aTGCs) is also presented in this paper, using both the $WV \rightarrow \ell \nu \mathrm {j}\mathrm {j}$ and $WV \rightarrow \ell \nu \mathrm {J}$ channels. Previous searches for charged aTGC contributions to $WV \rightarrow \ell \nu q q^{\prime }$ production have been conducted by the ATLAS Collaboration [14] using $7~\text {TeV}$ *pp* collisions, by the CMS Collaboration [15, 22] using 7 and $8~\text {TeV}$ *pp* collisions, and by the D0 [23] and CDF [24] collaborations using $p\bar{p}$ collisions. Most published aTGC searches in the $WV \rightarrow \ell \nu q q^{\prime }$ channel have reconstructed the $V \rightarrow q q^{\prime }$ as two small-*R* jets, with the exception of Ref. [22], which reconstructed the $V \rightarrow q q^{\prime }$ as a single large-*R* jet.

## Analysis overview

As mentioned above, measurements of $WV \rightarrow \ell \nu q q^{\prime }$ production are performed using either two small-*R* jets or a single large-*R* jet to reconstruct the hadronically decaying *V* boson. For both channels, the leptonically decaying *W* boson is reconstructed by requiring the presence of a lepton (electron or muon) and missing transverse momentum.

After applying stringent event selection requirements, the signal-to-background ratio remains quite low at 5–10$\%$, because of the large W + jets background. In order to distinguish the SM $WV$ signal from the background, the dijet mass distribution (in the $WV \rightarrow \ell \nu \mathrm {j}\mathrm {j}$ channel) or the mass distribution of the large-*R* jet (in the $WV \rightarrow \ell \nu \mathrm {J}$ channel) is used as a discriminating variable. The signal events peak near the *W* / *Z* mass in these distributions, while the shape of the dominant $W+\mathrm {jets}$ background is smoothly falling. In both channels, the signal is extracted from a fit to the discriminating variable. Wide fitting ranges are used, in order to allow the backgrounds to be constrained by the data.

A fiducial cross-section is measured separately in the $WV \rightarrow \ell \nu \mathrm {j}\mathrm {j}$ and the $WV \rightarrow \ell \nu \mathrm {J}$ channel; the fiducial phase spaces for the measurements are defined to be close to the experimental event selections. The fiducial cross-section in each channel is extracted from the previously mentioned fits. The events in the two channels partially overlap, because there are some events for which the $V \rightarrow q q^{\prime }$ decay can be reconstructed both as two small-*R* jets and as one large-*R* jet. In order to simplify the interpretation of the results and allow easier comparison with theoretical predictions, the overlap events are not removed, and both measurements are presented separately. No combination of the $WV \rightarrow \ell \nu \mathrm {j}\mathrm {j}$ and $WV \rightarrow \ell \nu \mathrm {J}$ cross-section measurements is performed. The electron and muon channels are combined when performing the measurements, since little improvement in sensitivity is expected from separating by lepton flavour. Event kinematics and the signal-to-background ratio are similar in the electron and muon channels, and the dominant sources of uncertainty are unrelated to lepton flavour.

A search for aTGC contributions is also performed in the $WV \rightarrow \ell \nu \mathrm {j}\mathrm {j}$ and $WV \rightarrow \ell \nu \mathrm {J}$ channels. The event selection is the same as for the cross-section measurements, except that a tighter requirement is made on the dijet mass or on the mass of the large-*R* jet. The search is performed by fitting the $p_{\mathrm {T}}$ distribution of the dijet system ($WV \rightarrow \ell \nu \mathrm {j}\mathrm {j}$ channel) or of the large-*R* jet ($WV \rightarrow \ell \nu \mathrm {J}$ channel). These distributions are sensitive to aTGCs, which are expected to lead to deviations from the SM prediction at high $p_{\mathrm {T}}$.

## ATLAS detector

The ATLAS detector [25], which surrounds one of the interaction points of the Large Hadron Collider (LHC) [26], is built of several subdetectors. The first subdetector layer consists of the inner detector (ID), which provides charged-particle tracking for $|\eta |<2.5$.^1^ The ID is further subdivided into (ordered from innermost to outermost) a pixel detector, a silicon-microstrip tracker, and a transition radiation tracker. Surrounding the ID there is a superconducting solenoid that provides a 2 T magnetic field. Outside of the solenoid, there is an electromagnetic (EM) calorimeter based on liquid-argon technology, which provides coverage up to $|\eta |=3.2$. Additionally, a scintillator-tile calorimeter provides hadronic energy measurements in the range $|\eta |<1.7$, and liquid-argon-based endcap and forward calorimeters extend the EM and hadronic measurements up to $|\eta |= 4.9$. A muon spectrometer, consisting of tracking and triggering detectors and three toroidal magnets, surrounds the calorimeters; it provides muon tracking and identification up to $|\eta |=2.7$ and triggering capability up to $|\eta |=2.4$.

A three-level trigger system is used to select the most interesting events for data storage [27]. An initial hardware-based trigger stage is followed by two software-based triggers, which reduce the final event rate to about 400 Hz.

## Data and Monte Carlo samples

This analysis is based on an integrated luminosity of $20.2\pm 0.4~\text{ fb }^{-1}$ of $8~\text {TeV}$ *pp* collisions recorded by the ATLAS detector in 2012. Events are required to pass one of several single-lepton triggers. The triggers require either an isolated electron or muon with $p_{\mathrm {T}}>24~\text {GeV}$, or an electron (muon) having $p_{\mathrm {T}}>60~(36)~\text {GeV}$ without an isolation requirement.

The nominal signal Monte Carlo (MC) samples consist of $q q^{\prime }\rightarrow WV$ events generated at next-to-leading order (NLO) in QCD using MC@NLO  v4.07 [28] interfaced with Herwig  v6.520 [29] and Jimmy  v4.31 [30] for the simulation of parton showering, hadronization, and the underlying event. The CT10 parton distribution function (PDF) set [31] and parameter values from the AUET2 tune [32] are used for these samples. The *W* and *Z* bosons are generated on-shell by MC@NLO and decayed subsequently by Herwig. The same MC configuration is also used to model aTGC contributions to *WV* production, using an event reweighting feature built into MC@NLO.

In order to study systematic uncertainties, alternative $q q^{\prime }\rightarrow WV$ samples are generated at NLO in QCD with Powheg-Box  [33–35] using the CT10 PDF set. The parton showering and hadronization is modelled with Pythia  8.175 [36] using the AU2 tune [37]. Off-shell *W* and $Z/\gamma ^{*}$ decays are included; the $Z/\gamma ^{*}$ decays have a requirement of $m_{q q^{\prime }}>20~\text {GeV}$ and $m_{\ell \ell }>20~\text {GeV}$.

Another set of alternative $q q^{\prime }\rightarrow WV$ samples are generated with Sherpa  v1.4.1 [38–41]. These samples are generated at leading order (LO) in QCD, but include up to three additional partons in the matrix element. Off-shell *W* and $Z/\gamma ^{*}$ decays are included; the $Z/\gamma ^{*}$ decays have a requirement of $m_{q q^{\prime }}>4~\text {GeV}$ and $m_{\ell \ell }>4~\text {GeV}$.

Contributions from $gg\rightarrow H\rightarrow WW^{*}$ are only at the $1\%$ level after applying the full event selection and are thus neglected. Signal MC samples for non-resonant $gg\rightarrow WW$ production are not used in the analysis, but the contribution from this process is estimated as described in Sect. 10, and included in the final cross-section predictions.

The $W+\mathrm {jets}$ and $Z+\mathrm {jets}$ backgrounds (collectively referred to as $V+\mathrm {jets}$) are modelled at LO in QCD with Sherpa  v1.4.1, with up to four additional final-state partons. The CT10 PDF set is used for these samples, and they are normalized using inclusive cross-sections that are next-to-next-to-leading order (NNLO) in QCD, obtained using FEWZ [42]. For studies of systematic uncertainties, alternative $W+\mathrm {jets}$ samples are generated with Alpgen  [43] interfaced with Pythia  6.426 [44], modelling the process at LO in QCD with up to five final-state partons. These additional samples use the Perugia 2011C tune [45] and the CTEQ6L1 PDF set [46].

The MC samples for the $t\bar{t}$ and single-top-quark (*t*-channel, *s*-channel, and *Wt*) processes (collectively referred to as top-quark processes) are generated with Powheg-Box  [47–49] interfaced with Pythia  6.426 [44] (or Pythia  6.427 for the *t*-channel single-top-quark process). All of these samples use the CT10 PDF set for the matrix element, the CTEQ6L1 PDF set for the parton shower, and the Perugia 2011C tune.

The *ZZ* background process is modelled with Powheg interfaced with Pythia  8. The sample is normalized using the NLO prediction from MCFM  [50, 51].

The MC samples are passed through a GEANT4-based [52] simulation of the ATLAS detector [53]. For some of the MC samples, a fast simulation is used that makes use of a parameterization of the showers in the calorimeter. The hard-scattering processes in the MC samples are overlaid with simulated minimum-bias events in order to model additional collisions in the same or neighbouring bunch crossings (“pile-up”). The MC samples are reweighted so that their pile-up profile matches that observed in the data.

## Event reconstruction

This analysis considers events with exactly one lepton (electron or muon), missing transverse momentum, and either two small-*R* jets or one large-*R* jet.

In each event, primary vertices are reconstructed, which must be formed from at least three tracks with $p_{\mathrm {T}}>400~\text {MeV}$. In case an event has multiple primary vertices (due to pile-up), the primary vertex with the highest $\sum p_{\mathrm {T}}^2$ of the associated tracks is defined as the hard-scatter vertex.

Electron candidates are formed from energy clusters in the EM calorimeter matched to ID tracks. They are required to have $p_{\mathrm {T}}>30~\text {GeV}$ and $|\eta |<2.47$. Candidates in the transition region between the barrel and endcaps of the EM calorimeter, $1.37<|\eta |<1.52$, are excluded. In order to ensure that the electron candidates are consistent with having been produced at the hard-scatter vertex, the transverse impact parameter $d_0$ and longitudinal impact parameter $z_0$ are required to satisfy $|d_{0}|/\sigma _{d_{0}}<5$ and $|z_0 \sin {\theta }|<0.5$ mm, respectively, where $\sigma _{d_{0}}$ is the uncertainty in the measured $d_0$. Both $d_0$ and $z_0$ are measured with respect to the hard-scatter vertex. Electron candidates must also satisfy the “tight” cut-based identification criteria from Ref. [54], based on track parameters and on the shower shapes in the calorimeter. Candidates must also pass isolation requirements based on calorimeter and track measurements. The calorimeter isolation requires $R^{\mathrm {iso}}_{\mathrm {cal}}<0.14$, where $R^{\mathrm {iso}}_{\mathrm {cal}}$ is defined as the scalar transverse energy sum of the calorimeter energy deposits within a $\Delta R\equiv \sqrt{(\Delta \eta )^2 + (\Delta \phi )^2}=0.3$ cone centred on the electron candidate (excluding transverse energy from the candidate itself), divided by the $p_{\mathrm {T}}$ of the electron candidate. Similarly, the track isolation requires $R^{\mathrm {iso}}_{\mathrm {ID}}<0.07$, where $R^{\mathrm {iso}}_{\mathrm {ID}}$ is the scalar sum of the $p_{\mathrm {T}}$ of the tracks within a $\Delta R=0.3$ cone centred on the electron candidate (excluding the $p_{\mathrm {T}}$ of the candidate’s track itself), divided by the electron candidate’s $p_{\mathrm {T}}$.

Muon candidates are formed from the combination of a track in the muon spectrometer and one in the ID. They are required to have $p_{\mathrm {T}}>30~\text {GeV}$ and $|\eta |<2.4$. Their impact parameters must satisfy $|d_{0}|/\sigma _{d_{0}}<3$ and $|z_0 \sin {\theta }|<0.5$ mm. The candidates must also satisfy the isolation criteria $R^{\mathrm {iso}}_{\mathrm {cal}}<0.07$ and $R^{\mathrm {iso}}_{\mathrm {ID}}<0.07$, where $R^{\mathrm {iso}}_{\mathrm {cal}}$ and $R^{\mathrm {iso}}_{\mathrm {ID}}$ are defined analogously to the electron case.

Small-*R* jets are reconstructed from topological energy clusters [55] in the calorimeter using the anti-$k_t$ algorithm [56] with radius parameter $R=0.4$. The jet energies are calibrated as described in Ref. [57] and are corrected for pile-up. They are required to have $p_{\mathrm {T}}>25~\text {GeV}$ and $|\eta |<2.5$ for the $WV \rightarrow \ell \nu \mathrm {j}\mathrm {j}$ channel. Small-*R* jets with $|\eta |<4.5$ are used in the $WV \rightarrow \ell \nu \mathrm {J}$ channel as part of a jet veto (see Sect. 6). In order to remove jets originating from pile-up, small-*R* jets having $p_{\mathrm {T}}<50~\text {GeV}$ and $|\eta |<2.4$ are required to have an absolute value of the “jet vertex fraction” variable (JVF) [58] greater than 0.5.

In the $WV \rightarrow \ell \nu \mathrm {J}$ channel, large-*R* jets are reconstructed using the anti-$k_t$ algorithm with radius parameter $R=1.0$, and are trimmed [20] using a subjet radius of 0.2 and a momentum-fraction parameter $f_\mathrm {cut}=0.05$; the trimming procedure discards soft subjets from the large-*R* jets and reduces their sensitivity to pile-up [21]. They are required to have $p_{\mathrm {T}}>200~\text {GeV}$ and $|\eta |<2.0$. The energies of the small-*R* and large-*R* jets and the masses of the large-*R* jets are calibrated using $p_{\mathrm {T}}$- and $\eta$-dependent scale factors [57, 59].

If an electron and a muon candidate share the same ID track, the electron candidate is rejected. If a small-*R* jet is within $\Delta R=0.2$ of a selected electron candidate, the jet is rejected; if the jet is within $0.2<\Delta R<0.4$ of a selected electron, the electron candidate is rejected. Muon candidates are rejected if they are within $\Delta R=0.4$ of a small-*R* jet. Finally, large-*R* jets are rejected if they are within $\Delta R=1.0$ of a selected lepton candidate. In the object selection stage, small-*R* jets and large-*R* jets are allowed to overlap; however, in the event selection stage a $\Delta R$ requirement is applied between the small-*R* and large-*R* jets, as explained in Sect. 6.

The missing transverse momentum $\vec {E}_{\text {T}}^{\text {miss}}$ is computed as the negative vector sum of the transverse momentum of all the detected objects in the event, including reconstructed jets, photons, electrons, and muons. An additional “soft term” is included that accounts for the $p_{\mathrm {T}}$ of clusters in the calorimeter which are not associated with any specific reconstructed object [60]. The magnitude of $\vec {E}_{\text {T}}^{\text {miss}}$ is denoted $E_{\text {T}}^{\text {miss}}$.

## Event selection

Two independent sets of event selection criteria are developed that target different event topologies: the $WV \rightarrow \ell \nu \mathrm {j}\mathrm {j}$ selection, described in Sect. 6.1, and the $WV \rightarrow \ell \nu \mathrm {J}$ selection, described in Sect. 6.2. The $WV \rightarrow \ell \nu \mathrm {J}$ channel and $WV \rightarrow \ell \nu \mathrm {j}\mathrm {j}$ channel differ significantly from one another in their kinematics, expected signal yields, and signal-to-background ratios. Therefore, the event selection criteria are optimized separately for the two channels.

For both the $WV \rightarrow \ell \nu \mathrm {j}\mathrm {j}$ and $WV \rightarrow \ell \nu \mathrm {J}$ selections, all events are required to contain at least one primary vertex. Events must have exactly one good electron or muon candidate. Events are vetoed if they contain any additional lepton candidates that have $p_{\mathrm {T}}>15~\text {GeV}$ and satisfy a looser set of selection criteria.

### $WV \rightarrow \ell \nu \mathrm {j}\mathrm {j}$ channel

Events must have $E_{\text {T}}^{\text {miss}} >40~\text {GeV}$ and a transverse mass^2^ $m_{\mathrm {T}}>40~\text {GeV}$. Events must contain exactly two small-*R* jets. The requirement of exactly two jets substantially reduces the background from top-quark decays. The pseudorapidity separation of the selected jets is required to satisfy $\Delta \eta (\mathrm {j},\mathrm {j})<1.5$, in order to improve the signal-to-background ratio.

In order to reduce the multijet background not removed by the $E_{\text {T}}^{\text {miss}} >40~\text {GeV}$ requirement, an azimuthal-angle difference between the $E_{\text {T}}^{\text {miss}}$ direction and the direction of the leading-$p_{\text {T}}$ jet of $|\Delta \phi (\mathrm {j}_1,E_{\text {T}}^{\text {miss}})| > 0.8$ is required. Also, both the $V \rightarrow q q^{\prime }$ and $W\rightarrow \ell \nu$ candidates must pass requirements on their transverse momenta: $p_{\mathrm {T}}(\mathrm {j}\mathrm {j})>100~\text {GeV}$ and $p_{\mathrm {T}}(W\rightarrow \ell \nu )>100~\text {GeV}$, where $p_{\mathrm {T}}(W\rightarrow \ell \nu )\equiv |\vec {E}_{\text {T}}^{\text {miss}} +\vec {p_{\mathrm {T}}}(\ell )|$. These $p_{\mathrm {T}}$ requirements enhance the separation between the signal and background distributions in the dijet mass.

As described in Sect. 8, the signal is extracted using a maximum-likelihood (ML) fit to the dijet mass ($m_{\mathrm {j}\mathrm {j}}$) distribution. In the dijet mass calculation, the mass of each individual jet is set to zero, which makes the variable easier to model in the MC simulation. Since the signal is extracted from a fit to $m_{\mathrm {j}\mathrm {j}}$, only a loose requirement is made on this variable: $40~\text {GeV}<m_{\mathrm {j}\mathrm {j}}<200~\text {GeV}$.

### $WV \rightarrow \ell \nu \mathrm {J}$ channel

Events must contain exactly one large-*R* jet with $p_{\mathrm {T}}>200~\text {GeV}$ and $|\eta |<2.0$. The backgrounds from top-quark decays are suppressed by rejecting events containing any small-*R* jets with $p_{\mathrm {T}}>25~\text {GeV}$ and $|\eta |<4.5$ that are separated from the large-*R* jet by $\Delta R(\mathrm {j},\mathrm {J})>1.0$. In order to suppress the multijet background, a requirement of $E_{\text {T}}^{\text {miss}} >50~\text {GeV}$ is applied. The trimmed mass of the large-*R* jet, $m_{\mathrm {J}}$, must be $50~\text {GeV}<m_{\mathrm {J}}<170~\text {GeV}$, and the signal is measured from the ML fit to $m_{\mathrm {J}}$.

Since the $WV \rightarrow \ell \nu \mathrm {j}\mathrm {j}$ and $WV \rightarrow \ell \nu \mathrm {J}$ event selections are done independently, some events pass both selections. About $10\%$ of the signal MC events that pass the $WV \rightarrow \ell \nu \mathrm {j}\mathrm {j}$ selection also pass the $WV \rightarrow \ell \nu \mathrm {J}$ selection, while about $50\%$ of the signal MC events that pass the $WV \rightarrow \ell \nu \mathrm {J}$ selection also pass the $WV \rightarrow \ell \nu \mathrm {j}\mathrm {j}$ selection.

## Background estimation

The methods for estimating the expected background yields and kinematic distributions are described in this section. The estimates from this section are used as inputs to the ML fit in which the signal is measured while the backgrounds are allowed to vary within their systematic uncertainties. In that ML fit, the $V+\mathrm {jets}$ normalization is allowed to vary without constraint, so the estimates given in this section are pre-fit estimates.

Most of the backgrounds ($W+\mathrm {jets}$, $Z+\mathrm {jets}$, $t\bar{t}$, single top-quark, and *ZZ*) are estimated using MC simulation, with data-driven corrections applied in some cases, as described later in this section. By far the largest background in the analysis is from $W+\mathrm {jets}$, followed by top-quark production. Despite the latter background’s subdominant contribution, it plays an important role because it contains contributions from real $W \rightarrow q q^{\prime }$ decays, which make it more difficult to distinguish from the signal. About $80\%$ of the top-quark background is due to $t\bar{t}$ production, and the remainder comes from single-top-quark production.

Multijet processes form another source of background. Multijet events can pass the event selection if they contain non-prompt leptons (produced from semileptonic decays of *c*- and *b*-hadrons) or “fake” leptons (resulting from misidentified jets). The multijet backgrounds are estimated using data-driven techniques, as described in Sects. 7.1 and 7.2.

### $WV \rightarrow \ell \nu \mathrm {j}\mathrm {j}$ channel

**Fig. 1:**
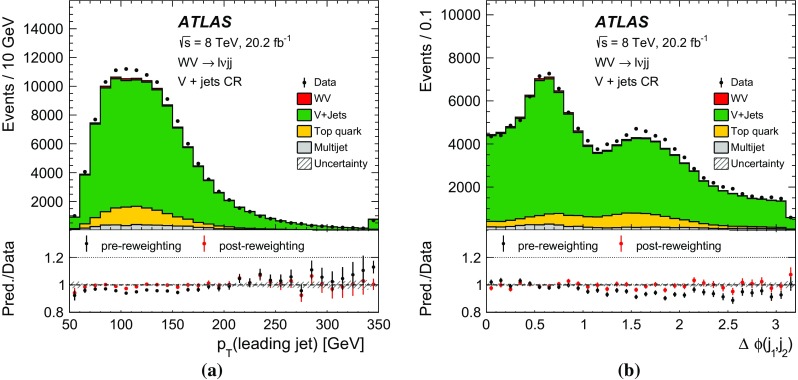
Comparisons between the data and the prediction in the $V+\mathrm {jets}$ control region of the $WV \rightarrow \ell \nu \mathrm {j}\mathrm {j}$ channel. The *top panel* shows the data and prediction before applying the $p_{\mathrm {T}}(\mathrm {j}_1)$ and $\Delta \phi (\mathrm {j}_1,\mathrm {j}_2)$ kinematic reweighting to the $V+\mathrm {jets}$ predictions. The distributions shown are **a** $p_{\mathrm {T}}$ of the leading jet and **b** $\Delta \phi$ between the leading jet and sub-leading jet. Overflow is included in the last bin of the $p_{\mathrm {T}}(\mathrm {j}_1)$ plot. The *bottom panel* shows the ratio of the SM prediction to the data before and after applying the kinematic reweighting to the $V+\mathrm {jets}$ prediction. The *hatched bands* indicate the statistical uncertainty in the predictions

The $V+\mathrm {jets}$ background prediction is MC-based, but data-driven corrections are applied to the MC prediction in order to improve the description of the jet kinematics. A $V+\mathrm {jets}$ control region (CR) is defined identically to the signal region, except that the region $65~\text {GeV}<m_{\mathrm {j}\mathrm {j}}<95~\text {GeV}$ is vetoed, in order to remove most of the signal events. One-dimensional reweighting functions of the variables $p_{\mathrm {T}}(\mathrm {j}_1)$ and $\Delta \phi (\mathrm {j}\mathrm {j})$ are derived from this $V+\mathrm {jets}$ CR. These reweighting functions have approximately $10\%$ effects on the shapes of the $p_{\mathrm {T}}(\mathrm {j}_1)$ and $\Delta \phi (\mathrm {j}\mathrm {j})$ distributions. Data–MC comparisons in the $V+\mathrm {jets}$ CR are shown in Fig. 1, before and after application of the reweighting functions. All further results in this paper are shown with these two reweighting functions applied to the $V+\mathrm {jets}$ MC samples. The same reweighting functions are used for both the $W+\mathrm {jets}$ and $Z+\mathrm {jets}$ processes. It was checked that the reweighting functions obtained from the low-$m_{\mathrm {j}\mathrm {j}}$ and high-$m_{\mathrm {j}\mathrm {j}}$ portions of the $V+\mathrm {jets}$ control region are compatible.

The top-quark background is modelled with MC simulation, and is cross-checked in a validation region containing three small-*R* jets, one of which is *b*-tagged using the MV1 algorithm [61, 62]. Good agreement is observed between the data and the MC simulation, so no corrections are applied to the prediction. The background from *ZZ* events is also modelled with MC simulation.

The data-driven multijet background estimate makes use of a multijet CR. The multijet CR is formed by selecting events in data that pass the same selection requirements as for the signal region, except that the lepton quality criteria are modified in order to produce a CR enriched in non-prompt and fake leptons. Lepton candidates satisfying these modified criteria are called “anti-identified” lepton candidates. Anti-identified muon candidates must have a non-negligible impact parameter, $|d_{0}|/\sigma _{d_{0}}>4$, and satisfy looser isolation criteria than the signal muon candidates. Anti-identified electrons must fail the “tight” but satisfy the “medium” cut-based identification criteria from Ref. [54], and are also required to contain a hit in the innermost layer of the pixel detector. In addition, the isolation criteria are modified for anti-identified electron candidates, in order to enrich the sample in non-prompt and fake electrons.

The shapes of the kinematic distributions [such as $m_{\mathrm {j}\mathrm {j}}$, $E_{\text {T}}^{\text {miss}}$, and $p_{\mathrm {T}}(\mathrm {j}\mathrm {j})$] of the multijet background are estimated from events in the multijet CR, after subtracting the MC predictions of the non-multijet contributions to the CR. These non-multijet contributions are about 20% (50%) of the total in the electron (muon) channel. The overall multijet background event yield is estimated from a fit to the $E_{\text {T}}^{\text {miss}}$ distribution of events that pass the full signal region selection, except that the requirements on $E_{\text {T}}^{\text {miss}}$ and $\Delta \phi (\mathrm {j}_1,E_{\text {T}}^{\text {miss}})$ (and also $\Delta \eta (\mathrm {j},\mathrm {j})$ and $m_{\mathrm {T}}$ for the muon channel) are removed in order to enhance the number of multijet events. This selection is referred to as the *extended* signal region. In this $E_{\text {T}}^{\text {miss}}$ fit, the multijet $E_{\text {T}}^{\text {miss}}$ shape is estimated from an extended multijet CR, defined analogously to the extended signal region, but requiring the lepton to pass the anti-identified-lepton selection. The $E_{\text {T}}^{\text {miss}}$ shapes of the other backgrounds are estimated using MC samples. The multijet event yield obtained from this fit is then extrapolated to the signal region, using the ratio of events in the multijet CR and the extended multijet CR, corrected for non-multijet contributions. The multijet background estimates are performed separately for the electron and muon channels. Only about $5\%$ of the total multijet background is in the muon channel.

**Fig. 2:**
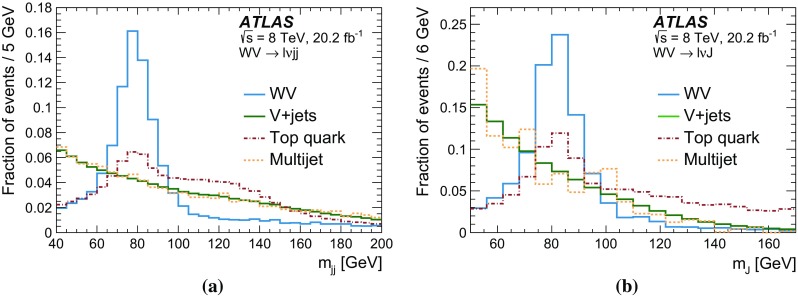
The shapes of **a** the predicted $m_{\mathrm {j}\mathrm {j}}$ distributions in the $WV \rightarrow \ell \nu \mathrm {j}\mathrm {j}$ signal region and **b** the predicted $m_{\mathrm {J}}$ distributions in the $WV \rightarrow \ell \nu \mathrm {J}$ signal region, for the signal (peaked near 80 $\text {GeV}$) and various background processes. The distributions are normalized to unity

The expected signal and background yields in the $WV \rightarrow \ell \nu \mathrm {j}\mathrm {j}$ signal region are given in Table 1, and compared to the number of events observed in data. The predictions for the $m_{\mathrm {j}\mathrm {j}}$ distribution shapes of the signal and backgrounds are shown in Fig. 2a.

**Table 1:** Expected number of signal and background events in the $WV \rightarrow \ell \nu \mathrm {j}\mathrm {j}$ and $WV \rightarrow \ell \nu \mathrm {J}$ signal regions, prior to performing the $m_{\mathrm {j}\mathrm {j}}$ and $m_{\mathrm {J}}$ fits. The quoted uncertainties only include detector-related uncertainties and statistical uncertainties of the MC samples and control regions. The number of events observed in data is also shown. The signal predictions only correspond to $q q^{\prime }$-initiated *WV* production

	$WV \rightarrow \ell \nu \mathrm {j}\mathrm {j}$	$WV \rightarrow \ell \nu \mathrm {J}$
Signal		
*WW*	2860 ± 110	542 ± 61
*WZ*	730 ± 30	128 ± 15
Total expected signal	3590 ± 140	670 ± 75
Background		
$W+\mathrm {jets}$	136,000 ± 8600	10500 ± 1300
$Z+\mathrm {jets}$	2750 ± 340	245 ± 32
$t\bar{t}$	12,980 ± 520	1130 ± 150
Single top-quark	3620 ± 150	249 ± 35
Multijet	3689 ± 60	313 ± 18
*ZZ*	14 ± 1	–
Total expected background	159,000 ± 8600	12,400 ± 1500
Total SM expected	162,600 ± 8700	13,100 ± 1600
Observed	164,502	12,999
*S* / *B* ($65~\text {GeV}<m_{\mathrm {j}\mathrm {j}}<95~\text {GeV}$)	$5.5\%$	$10.1\%$
$S/\sqrt{B}$ ($65~\text {GeV}<m_{\mathrm {j}\mathrm {j}}<95~\text {GeV}$)	11.1	7.1

### $WV \rightarrow \ell \nu \mathrm {J}$ channel

In the $WV \rightarrow \ell \nu \mathrm {J}$ channel, the $W+\mathrm {jets}$, $Z+\mathrm {jets}$, and top-quark backgrounds are estimated using MC samples. The MC predictions for the two largest backgrounds ($W+\mathrm {jets}$ and top-quark production) are corrected by scale factors obtained from dedicated control regions.

**Fig. 3:**
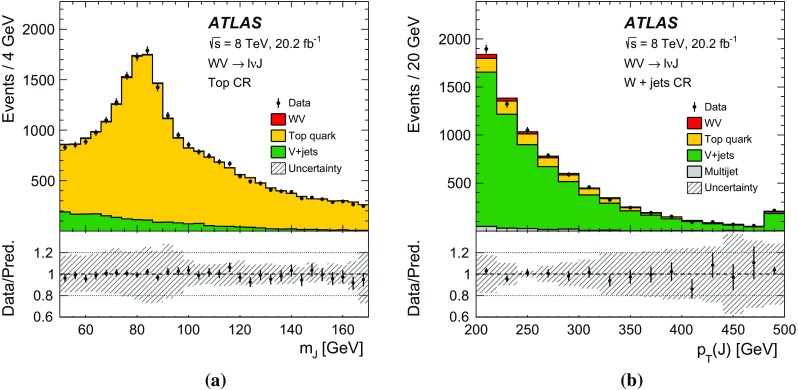
Comparison between data and prediction in the $WV \rightarrow \ell \nu \mathrm {J}$ channel for **a** $m_{\mathrm {J}}$ in the top CR, and **b** $p_{\mathrm {T}}(\mathrm {J})$ in the $W+\mathrm {jets}$ CR. A scale factor is applied to the top-quark background prediction in the top CR and the $W+\mathrm {jets}$ CR, and a scale factor is applied to the $W+\mathrm {jets}$ background prediction (which is part of the “$V+\mathrm {jets}$” histogram) in the $W+\mathrm {jets}$ CR. The *hatched bands* indicate the systematic uncertainty of the prediction. For the $V+\mathrm {jets}$ component, only shape systematic uncertainties are included in the bands

The top-quark control region (top CR) is formed by events satisfying the signal region selection, except that the presence of at least one small-*R* *b*-tagged jet with $p_{\mathrm {T}}>25~\text {GeV}$ and $\Delta R(\mathrm {j},\mathrm {J})>1.0$ is required instead of applying the nominal veto on small-R jets. The jets are *b*-tagged using the MV1 algorithm [61, 62], using a working point with a *b*-tagging efficiency of about 70% and a gluon/light-quark jet rejection factor of over 100 in $t\bar{t}$ events. About $90\%$ of the events in this top CR originate from top-quark backgrounds. There is a deficit in data in the top CR relative to the MC prediction, which is attributed to a mismodelling of the top-quark backgrounds. A global scale factor of $0.87$ for the top-quark backgrounds is obtained from this CR, after subtracting the prediction for non-top-quark backgrounds. The data in the top CR is shown in Fig. 3a, compared to the SM prediction after application of the top-quark scale factor. This scale factor is applied to the top-quark background predictions in the signal region.

The control region for the $W+\mathrm {jets}$ background ($W+\mathrm {jets}$ CR) is obtained by applying the standard signal region selection, but adding the requirement that $m_{\mathrm {J}}<65~\text {GeV}$ or $m_{\mathrm {J}}>95~\text {GeV}$. This additional $m_{\mathrm {J}}$ requirement removes almost all of the *WV* signal events and also a large fraction of the top-quark events. About $85\%$ of the events in this CR originate from $W+\mathrm {jets}$ backgrounds. The top-quark background prediction in the $W+\mathrm {jets}$ CR is scaled by the top-quark scale factor obtained above. A data deficit is observed in the $W+\mathrm {jets}$ CR relative to the prediction. A global scale factor of $0.84$ is obtained for the $W+\mathrm {jets}$ background, after subtracting the expected contributions from the other signal/background processes. A comparison between the data and the prediction in the $W+\mathrm {jets}$ CR is shown in Fig. 3b, after application of the $W+\mathrm {jets}$ scale factor. The $W+\mathrm {jets}$ scale factor is applied to the $W+\mathrm {jets}$ prediction in the signal region.

The method for estimating the multijet background is similar to that used in the $WV \rightarrow \ell \nu \mathrm {j}\mathrm {j}$ channel. As in the $WV \rightarrow \ell \nu \mathrm {j}\mathrm {j}$ channel, a multijet CR is defined by requiring an “anti-identified” lepton candidate. The shapes of the kinematic distributions are estimated from this CR using the same method as in the $WV \rightarrow \ell \nu \mathrm {j}\mathrm {j}$ channel. The non-multijet background contributions to the CR are about 6% of the total. The multijet event yield is estimated from a fit to the $E_{\text {T}}^{\text {miss}}$ distribution, as in the $WV \rightarrow \ell \nu \mathrm {j}\mathrm {j}$ channel, but the only requirement that is removed for the definiton of the extended signal region/multijet CR is the $E_{\text {T}}^{\text {miss}} >50~\text {GeV}$ requirement. The multijet background is found to be negligible for the muon channel, so only the contribution in the electron channel is considered for the final results.

The numbers of expected and observed events in the $WV \rightarrow \ell \nu \mathrm {J}$ signal region are summarized in Table 1. The previously mentioned top-quark and $W+\mathrm {jets}$ scale factors are applied to the predictions. The contribution from *ZZ* events is expected to be very small in the $WV \rightarrow \ell \nu \mathrm {J}$ channel, so it is neglected. The nominal predictions for the $m_{\mathrm {J}}$ distribution shapes of the signal and backgrounds are shown in Fig. 2b.

## Cross-section extraction

The fiducial cross-section $\sigma _{\mathrm {fid}}$ for $WV \rightarrow \ell \nu q q^{\prime }$ production is measured independently for the $WV \rightarrow \ell \nu \mathrm {j}\mathrm {j}$ and $WV \rightarrow \ell \nu \mathrm {J}$ phase spaces, in both cases using the formula:

$$\begin{aligned} \sigma _{\mathrm {fid}}= & {} \frac{N^{WV}}{\mathcal {L}\cdot D_{\mathrm {fid}}} \,, \end{aligned}$$

where $N^{WV}$ is the measured signal yield, $\mathcal {L}$ is the integrated luminosity, and $D_{\mathrm {fid}}$ is a factor that corrects for experimental acceptance and efficiencies. Since this analysis measures $N^{WV}$ as the sum of the *WW* and *WZ* processes, which can each have different acceptances and efficiencies, $D_{\mathrm {fid}}$ is given by:

$$\begin{aligned} D_{\mathrm {fid}}= f^{WW}_{\mathrm {fid}}\cdot C^{WW}+ \left( 1-f^{WW}_{\mathrm {fid}}\right) \cdot C^{WZ}, \end{aligned}$$

where the $C^{WV}$ are reconstruction correction factors and the variable $f^{WW}_{\mathrm {fid}}$ is the predicted ratio of the *WW* fiducial cross-section to the $WW+WZ$ fiducial cross-section. The $C^{WV}$ and $f^{WW}_{\mathrm {fid}}$ values are estimated from MC simulation. The $C^{WV}$ factors are defined as the predicted number of *WV* signal events passing the reconstruction-level event selection divided by the number of *WV* events in the fiducial phase space defined with generator-level particles. The $C^{WV}$ factors account for reconstruction inefficiencies, resolution effects, and for contributions to the signal region from *WV* events that do not decay to $\ell \nu q q^{\prime }$ (such as $WV \rightarrow \tau \nu q q^{\prime }$ or $WW \rightarrow \ell \nu \ell \nu$); the latter are included in the $C^{WV}$ numerator and not in the denominator. The cross-section $\sigma _{\mathrm {fid}}$ is measured for the sum of the electron and muon channels, so $D_{\mathrm {fid}}$ is computed as a weighted average over the electron and muon channels. The fiducial cross-section measurement therefore assumes that the signal MC simulation correctly predicts the ratio of *WW* to *WZ* and of electrons to muons. The value of $D_{\mathrm {fid}}$ is $0.83 \pm 0.05$ in the $WV \rightarrow \ell \nu \mathrm {j}\mathrm {j}$ channel and $0.60 \pm 0.08$ in the $WV \rightarrow \ell \nu \mathrm {J}$ channel, including systematic uncertainties (see Sect. 9).

The fiducial phase spaces for the $WV \rightarrow \ell \nu \mathrm {j}\mathrm {j}$ and $WV \rightarrow \ell \nu \mathrm {J}$ channels are defined in Sects. 8.1 and 8.2, respectively. These fiducial phase spaces partially overlap. In order to cope with the small signal-to-background ratios in this analysis (5–10$\%$), the cross-section $\sigma _{\mathrm {fid}}$ is extracted using a binned ML fit to the $m_{\mathrm {j}\mathrm {j}}$ distribution (in the $WV \rightarrow \ell \nu \mathrm {j}\mathrm {j}$ analysis) or the $m_{\mathrm {J}}$ distribution (in the $WV \rightarrow \ell \nu \mathrm {J}$ analysis). The ML fits are performed on the sum of the electron and muon channels. It was cross-checked that the electron and muon channels are compatible, in both the $WV \rightarrow \ell \nu \mathrm {j}\mathrm {j}$ and $WV \rightarrow \ell \nu \mathrm {J}$ channels.

In the ML fits, the value of $\sigma _{\mathrm {fid}}$ and the $V+\mathrm {jets}$ background yield are both free to vary without constraint. Systematic uncertainties in the signal and backgrounds are incorporated in the fit by including nuisance parameters that are allowed to vary within prior constraints. The nuisance parameters allow the luminosity, $D_{\mathrm {fid}}$, the non-$V+\mathrm {jets}$ background yields, and the $m_{\mathrm {j}\mathrm {j}}$ and $m_{\mathrm {J}}$ shapes of the signal and background distributions to vary within their systematic uncertainties. The correlations between the uncertainty in $D_{\mathrm {fid}}$ and the uncertainty in the signal $m_{\mathrm {j}\mathrm {j}}/m_{\mathrm {J}}$ shapes are accounted for in the fit. The sources of systematic uncertainty and the methods to assess these uncertainties are described in detail in Sect. 9.

### $WV \rightarrow \ell \nu \mathrm {j}\mathrm {j}$ fiducial phase space

The $WV \rightarrow \ell \nu \mathrm {j}\mathrm {j}$ fiducial phase space is defined to closely match the experimental event selection. The phase-space definition requires a *WV* pair with the bosons decaying as $V \rightarrow q q^{\prime }$ and $W\rightarrow \ell \nu$, where $\ell =e,\mu$. Events containing other kinds of *WV* decay channels (such as $WW\rightarrow \ell \nu \ell \nu$ events or $WV \rightarrow \tau \nu q q^{\prime }$ with the $\tau$ decaying to $\ell +X$), are not included in the fiducial phase-space definition. Such *WV* events can still pass the experimental event selection (where they are included in the signal category), and they are accounted for in the $D_{\mathrm {fid}}$ definition.

Leptons selected in the fiducial region must have $p_{\mathrm {T}}(\ell )>30~\text {GeV}$ and $|\eta (\ell )|<2.47$. The four-momentum of the lepton is modified by adding to it the four-momenta of all the photons within $\Delta R=0.1$, excluding photons produced by hadron decays. Particle-level anti-$k_t$ $R=0.4$ jets are constructed using as constituents all stable particles, excluding muons and neutrinos. Stable particles are defined as those having a mean lifetime of $\tau >30$ ps. The particle-level jets must have $p_{\mathrm {T}}>25~\text {GeV}$ and $|\eta |<2.5$. Jets within $\Delta R=0.2$ of a selected electron are rejected, and then leptons within $\Delta R=0.4$ of a remaining jet are rejected. The true $E_{\text {T}}^{\text {miss}}$ in the event is defined as the magnitude of the vector $p_{\mathrm {T}}$ sum of all the neutrinos.

The event must have exactly one lepton and two $R=0.4$ jets matching the above definitions. The remaining requirements for the fiducial phase space are summarized in Table 2, and are analogous to the experimental event selection, but are defined using the lepton, $E_{\text {T}}^{\text {miss}}$, and particle-level jets described in this section.

**Table 2:** Summary of the fiducial phase-space definitions. All the specified selection criteria are applied at the particle level as specified in the text. The notations “$\mathrm {j}$” and “$\mathrm {J}$” refer to $R=0.4$ and $R=1.0$ jets, respectively, as explained in the text

	$WV \rightarrow \ell \nu \mathrm {j}\mathrm {j}$	$WV \rightarrow \ell \nu \mathrm {J}$
Lepton	$N_{\ell }=1$ with $p_{\mathrm {T}}>30~\text {GeV}$ and $|\eta |<2.47$,	
	$$\Delta R(\ell ,\mathrm {j})>0.4$$	
$W\rightarrow \ell \nu$	$p_{\mathrm {T}}(\ell \nu )>100~\text {GeV}$	−
	$m_{\mathrm {T}}>40~\text {GeV}$	−
$E_{\text {T}}^{\text {miss}}$	$E_{\text {T}}^{\text {miss}} > 40~\text {GeV}$	$E_{\text {T}}^{\text {miss}} > 50~\text {GeV}$
Jet	$N_{\mathrm {j}}=2$ with $p_{\mathrm {T}}>25~\text {GeV}$, $|\eta |<2.5$,	$N_{\mathrm {J}}=1$ with $p_{\mathrm {T}}>200~\text {GeV}$, $|\eta |<2.0$,
	$\Delta R(\mathrm {j},e)>0.2$	$\Delta R(\mathrm {J},\ell )>1.0$
		No small-*R* jets with $p_{\mathrm {T}}>25~\text {GeV}$, $|\eta |<4.5$,
		$\Delta R(\mathrm {j},\mathrm {J})>1.0$, $\Delta R(\mathrm {j},e)>0.2$
	$40<m_{\mathrm {j}\mathrm {j}}<200~\text {GeV}$	$50<m_{\mathrm {J}}<170~\text {GeV}$
	$p_{\mathrm {T}}(\mathrm {j}\mathrm {j})>100~\text {GeV}$	−
	$\Delta \eta (\mathrm {j},\mathrm {j})<1.5$	−
Global	$\Delta \phi (\mathrm {j}_1,E_{\text {T}}^{\text {miss}})>0.8$	−

### $WV \rightarrow \ell \nu \mathrm {J}$ fiducial phase space

As in the $WV \rightarrow \ell \nu \mathrm {j}\mathrm {j}$ channel, the fiducial phase-space definition requires a *WV* pair with $V \rightarrow q q^{\prime }$ and $W\rightarrow \ell \nu$. Leptons, $E_{\text {T}}^{\text {miss}}$, and particle-level $R=0.4$ jets are defined in the same way as in the $WV \rightarrow \ell \nu \mathrm {j}\mathrm {j}$ channel, except that two sets of leptons and small-*R* jets are considered: *central* leptons (small-*R* jets) are required to have $|\eta |<2.47$ ($|\eta |<2.5$), and *extended* leptons and small-*R* jets are required to have $|\eta |<4.5$. Particle-level large-*R* jets are defined by applying the anti-$k_t$ algorithm with radius parameter $R=1.0$ to all stable particles, excluding muons and neutrinos. No trimming is applied to these jets. The large-*R* jets are required to have $p_{\mathrm {T}}>200~\text {GeV}$ and $|\eta |<2.0$. Central (extended) small-*R* jets that are within $\Delta R=0.2$ of a central (extended) electron are rejected. Then, central leptons are rejected if they are within $\Delta R=0.4$ of a remaining central small-*R* jet. Large-*R* jets are rejected if they are within $\Delta R=1.0$ of any remaining central leptons. Events are required to contain exactly one central lepton and one large-*R* jet with the above definitions, and events are discarded if they contain any extended small-*R* jets with $\Delta R(\mathrm {j},\mathrm {J})>1.0$. The event must also have $E_{\text {T}}^{\text {miss}} >50~\text {GeV}$, and the large-*R* jet must have a mass greater than $50~\text {GeV}$. The fiducial phase-space definition is summarized in Table 2.

## Systematic uncertainties

Systematic uncertainties in the measured $\sigma _{\mathrm {fid}}$ can be due to uncertainties in $\mathcal {L}$, $D_{\mathrm {fid}}$, and/or $N^{WV}$. Uncertainties in the measured $N^{WV}$ can in turn be due to uncertainties in the background yields or in the shapes of the kinematic distributions ($m_{\mathrm {j}\mathrm {j}}$, $m_{\mathrm {J}}$) of the signal and backgrounds (hereafter called “shape uncertainties”). The dominant systematic uncertainties in the $\sigma _{\mathrm {fid}}$ measurement are those affecting the measured $N^{WV}$.

A wide variety of detector-related experimental uncertainties are considered, which affect $D_{\mathrm {fid}}$, the predicted background yields, and the signal and background shapes. The most important of these uncertainties are those related to the jet reconstruction. Uncertainties in the small-*R* jet energy scale and resolution are accounted for [57, 63]. In the $WV \rightarrow \ell \nu \mathrm {J}$ channel, uncertainties in the large-*R* jet energy and jet mass scales are also taken into account. The scale uncertaities of the large-*R* jets are estimated using a double-ratio method that compares calorimeter- and track-jets in data and MC simulation [21]. The energy and mass resolution uncertainties of large-*R* jets are estimated by smearing the jet energies/masses so as to degrade the resolutions by 20%; this approach is based on prior studies of large-*R* jets [64, 65]. The systematic uncertainty due to the JVF requirement is also included [66]. In addition to the jet-related uncertainties, there are also systematic uncertainties in the electron and muon reconstruction (including triggering, object reconstruction, identification, and the energy scale and resolution) [54, 67–70]. The effects of the jet and lepton uncertainties are propagated to the $E_{\text {T}}^{\text {miss}}$ calculation, and an additional systematic uncertainty in the soft terms entering the $E_{\text {T}}^{\text {miss}}$ calculation is also included [60].

In the cross-section fits, the $V+\mathrm {jets}$ yield is taken to be a free parameter, while several uncertainties in the modelling of its shape are accounted for (in addition to the shape uncertainties from the previously mentioned detector effects). Systematic uncertainties in the $V+\mathrm {jets}$ shape are estimated by varying the MC event generator used (Sherpa compared to Alpgen+Pythia). The differences between the predictions of the two generators are taken as additional systematic uncertainties. Additional uncertainties in the $V+\mathrm {jets}$ shape are estimated by varying the renormalization and factorization scales by factors of 2 and 0.5, and by varying the scale used in Sherpa for matching the matrix elements to the parton showers [39] from its nominal value of $20~\text {GeV}$ to alternative values of $15~\text {GeV}$ and $30~\text {GeV}$. In the $WV \rightarrow \ell \nu \mathrm {j}\mathrm {j}$ channel, the uncertainty in the shapes of the $V+\mathrm {jets}$ predictions due to the two kinematic reweighting functions (see Sect. 7.1) is estimated by including the full difference between applying and not applying each reweighting function as additional systematic uncertainties. In the $WV \rightarrow \ell \nu \mathrm {j}\mathrm {j}$ channel, an uncertainty of $10\%$ in the $(W+\mathrm {jets})/(Z+\mathrm {jets})$ cross-section ratio is also included; this uncertainty is ignored in the $WV \rightarrow \ell \nu \mathrm {J}$ channel as it has a negligible effect.

For the $t\bar{t}$ background, uncertainties due to the matrix-element event generator, parton shower/hadronization model, and amount of initial- and final-state radiation are all included. The theoretical uncertainties in the top-quark background cross-sections are also taken into account. In the $WV \rightarrow \ell \nu \mathrm {J}$ channel, instead of using the theoretical cross-section uncertainty, the top-quark background is assigned a normalization uncertainty of $14\%$ to account for the uncertainty in the data-driven scale factor. Systematic uncertainties in the multijet background estimate are also included, which affect both its normalization and its shape. These uncertainties are derived from studies of variations of the data-driven estimate, such as changing the control region definitions and varying the non-multijet background subtraction. The uncertainty in the multijet yield amounts to $30\%$ ($100\%$) for the electron (muon) channel in the $WV \rightarrow \ell \nu \mathrm {j}\mathrm {j}$ channel. In the $WV \rightarrow \ell \nu \mathrm {J}$ channel, an uncertainty of $50\%$ is assigned to the multijet yield in the electron channel, while the multijet background is neglected in the muon channel. A $30\%$ uncertainty is assigned to the *ZZ* event yield in the $WV \rightarrow \ell \nu \mathrm {j}\mathrm {j}$ channel, to account for uncertainties in the *ZZ* cross-section and the extrapolation to the fiducial phase space.

Additionally, the uncertainty in the modelling of pile-up interactions is accounted for [71]. The uncertainty in the integrated luminosity is also included, computed as described in Ref. [72]. The statistical uncertainty of the MC samples is taken into account, which affects each bin in the ML fits in an uncorrelated way.

Uncertainties in the signal shapes and in the $D_{\mathrm {fid}}$ parameter due to variations of the signal model are computed by varying the renormalization and factorization scales by factors of 2 and 0.5, and by comparing the nominal MC@NLO signal samples to alternative samples generated with Sherpa and Powheg +Pythia 8. The effect on $D_{\mathrm {fid}}$ from the uncertainties in the CT10 PDF set is also taken into account; the PDF uncertainty has a negligible impact on the signal shapes.

The measured $\sigma _{\mathrm {fid}}$ values are compared to theoretical predictions from MC@NLO. The uncertainty in the theoretical $\sigma _{\mathrm {fid}}$ prediction is calculated including the uncertainties due to renormalization and factorization scales. Since the fiducial phase spaces contain a veto on additional jets, the Stewart–Tackmann procedure [73] is used to estimate the scale uncertainties. These uncertainties are also propagated to the theoretical $f^{WW}_{\mathrm {fid}}$ value which enters into the $D_{\mathrm {fid}}$ calculation, although the effect of this on the measured $\sigma _{\mathrm {fid}}$ is very small ($\sim$0.1%). PDF-induced uncertainties in the theoretical prediction are also taken into account.

## Cross-section results

**Fig. 4:**
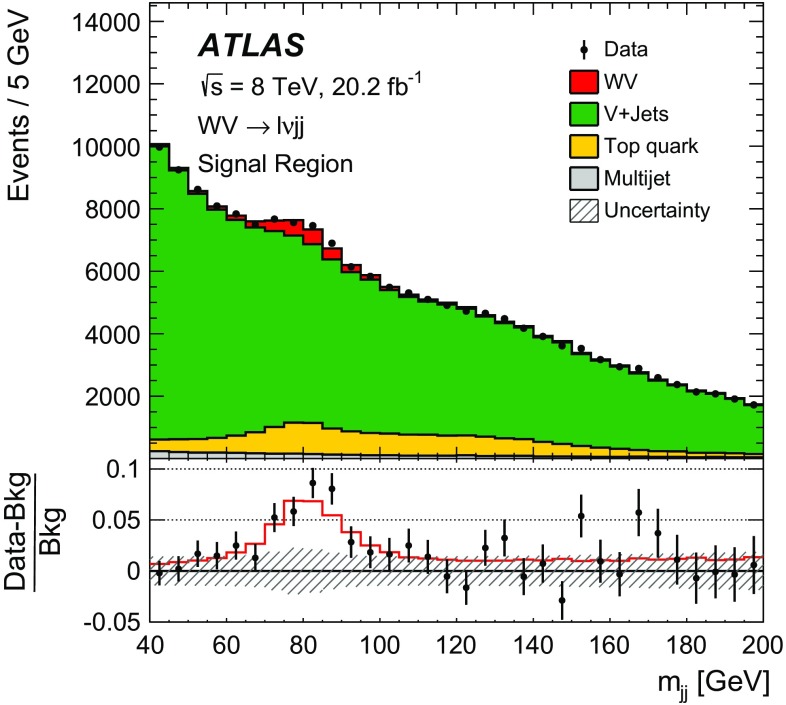
The observed $m_{\mathrm {j}\mathrm {j}}$ distribution in the $WV \rightarrow \ell \nu \mathrm {j}\mathrm {j}$ signal region, overlaid with the post-fit background and signal estimates. The *hatched band* indicates the total uncertainty of the fit result

The result of the ML fit to the $m_{\mathrm {j}\mathrm {j}}$ distribution for the $WV \rightarrow \ell \nu \mathrm {j}\mathrm {j}$ channel is shown in Fig. 4. The fit is performed on the sum of events in the electron and muon channels. The observed significance is $4.5\sigma$, including statistical and systematic uncertainties,^3^ while the expected significance, calculated using the Asimov data set [74], is $5.2\sigma$. The fitted $V+\mathrm {jets}$ background normalization is $1.02\pm 0.01$ times its pre-fit value, while the fitted top-quark background normalization is $0.96\pm 0.10$ times its pre-fit value.

The fiducial cross-section for the signal process is extracted from the fit as described in Sect. 8, and the result is

$$\begin{aligned} \sigma _{\mathrm {fid}}(WV \rightarrow \ell \nu \mathrm {j}\mathrm {j},\text {observed})= & {} 209 \pm 28 (\text {stat}) \pm 45 (\text {syst})~\text {fb}. \end{aligned}$$

The impacts of the various systematic uncertainties on the cross-section measurement are shown in Table 3. The measurement can be compared to the theoretical prediction of

$$\begin{aligned} \sigma _{\mathrm {fid}}(WV \rightarrow \ell \nu \mathrm {j}\mathrm {j},\text {theory})= & {} 225 \pm 13~\text {fb}\,. \end{aligned}$$

The theoretical prediction is obtained using MC@NLO for the $q q^{\prime }\rightarrow WV$ prediction. The $gg\rightarrow WW$ prediction is also included, and is calculated using the total NLO $gg\rightarrow WW$ cross-section prediction [75] multiplied by the $q q^{\prime }\rightarrow WW$ acceptance from MC@NLO. The $gg\rightarrow WW$ contribution increases the fiducial cross-section prediction by $4\%$ in both the $WV \rightarrow \ell \nu \mathrm {j}\mathrm {j}$ and $WV \rightarrow \ell \nu \mathrm {J}$ channels. Given the relatively small $gg\rightarrow WW$ contribution, the possible differences in acceptance between the $gg\rightarrow WW$ and $q q^{\prime }\rightarrow WW$ processes are neglected. The uncertainty in the MC@NLO prediction is described in Sect. 9.

**Table 3:** Breakdown of the uncertainties in the measured fiducial cross-section in the $WV \rightarrow \ell \nu \mathrm {j}\mathrm {j}$ channel. Uncertainties smaller than 1% are omitted from the table

Source of uncertainty	Relative uncertainty for $\sigma _{\mathrm {fid}}$ (%)
Top-quark background modelling	13
Signal modelling	12
$V+\mathrm {jets}$ modelling	4
Multijet background modelling	1
Small-*R* jet energy/resolution	9
Other experimental (leptons, pile-up)	4
Luminosity	2
MC statistics	9
Data statistics	14

**Fig. 5:**
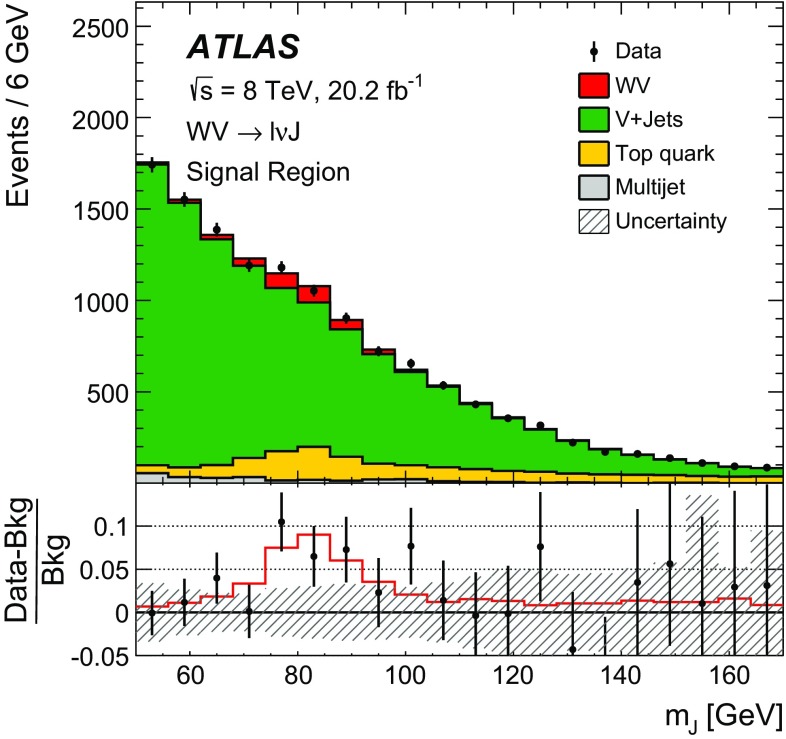
The observed $m_{\mathrm {J}}$ distribution in the $WV \rightarrow \ell \nu \mathrm {J}$ signal region, overlaid with the post-fit background and signal estimates. The *hatched band* indicates the total uncertainty of the fit result

The result of the $m_{\mathrm {J}}$ fit for the $WV \rightarrow \ell \nu \mathrm {J}$ channel is shown in Fig. 5. Although the signal-to-background ratio is better in this case than in the $WV \rightarrow \ell \nu \mathrm {j}\mathrm {j}$ channel, the total number of signal events is much smaller. The observed significance of the result is $1.3\sigma$ (including statistical and systematic uncertainties), compared to an expected significance of $2.5\sigma$. The fitted $V+\mathrm {jets}$ (top-quark) background normalization is $1.01\pm 0.04$ ($1.06\pm 0.20$) times its pre-fit value.

The extracted fiducial cross-section for the signal process is

$$\begin{aligned} \sigma _{\mathrm {fid}}(WV \rightarrow \ell \nu \mathrm {J},\text {observed})= & {} 30 \pm 11 (\text {stat}) \pm 22 (\text {syst})~\text {fb}, \end{aligned}$$

which is compatible with the theoretical prediction of

$$\begin{aligned} \sigma _{\mathrm {fid}}(WV \rightarrow \ell \nu \mathrm {J},\text {theory})= & {} 58 \pm 15~\text {fb}. \end{aligned}$$

The breakdown of the uncertainties contributing to the fiducial cross-section measurement is shown in Table 4.

**Table 4:** Breakdown of the uncertainties in the measured fiducial cross-section in the $WV \rightarrow \ell \nu \mathrm {J}$ channel. Uncertainties smaller than 1% are omitted from the table

Source of uncertainty	Relative uncertainty for $\sigma _{\mathrm {fid}}$ (%)
$V+\mathrm {jets}$ modelling	60
Top-quark background modelling	32
Signal modelling	15
Multijet background modelling	13
Large-*R* jet energy/resolution	45
Small-*R* jet energy/resolution	16
Other experimental (leptons, pile-up)	3
Luminosity	2
MC statistics	19
Data statistics	33

**Fig. 6:**
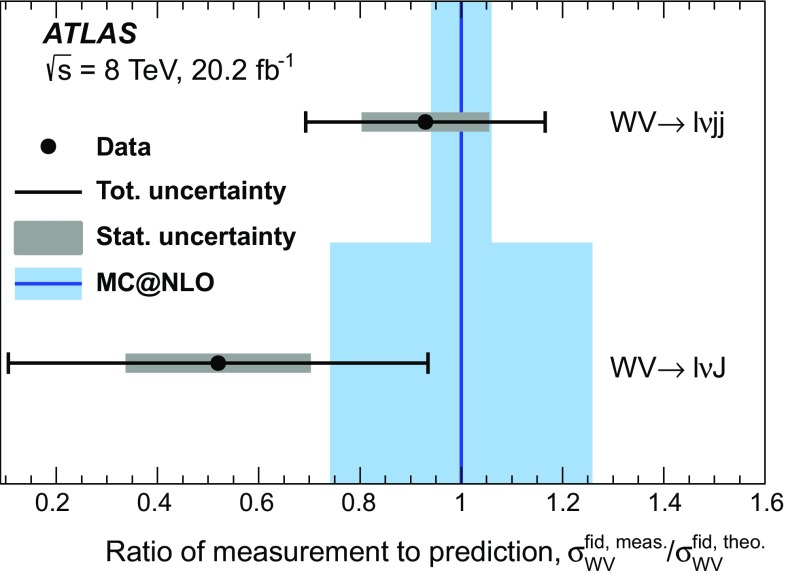
The ratios of the measured fiducial cross-sections to the cross-sections predicted by MC@NLO, for the $WV \rightarrow \ell \nu \mathrm {j}\mathrm {j}$ and $WV \rightarrow \ell \nu \mathrm {J}$ phase spaces. The $WV \rightarrow \ell \nu \mathrm {j}\mathrm {j}$ and $WV \rightarrow \ell \nu \mathrm {J}$ phase spaces partially overlap

The cross-section measurements are summarized in Fig. 6. As mentioned in Sect. 8, the two cross-section measurements are performed in partially overlapping phase spaces. The uncertainty in the theory prediction is significantly larger in the $WV \rightarrow \ell \nu \mathrm {J}$ channel than in the $WV \rightarrow \ell \nu \mathrm {j}\mathrm {j}$ channel. The theoretical uncertainty in the $WV \rightarrow \ell \nu \mathrm {J}$ channel is dominated by the scale uncertainties, which are particularly large because of the aggressive jet veto in this channel (only about 30% of signal MC events pass the jet veto in the $WV \rightarrow \ell \nu \mathrm {J}$ channel, compared to about 80% in the $WV \rightarrow \ell \nu \mathrm {j}\mathrm {j}$ channel).

## Constraints on anomalous gauge couplings

In many extensions of the SM, diboson production can be modified, such as through new resonances that couple to bosons. If the scale of new physics is sufficiently high, new resonances may not be visible in the current data; however, diboson production could still be affected below the new-physics scale, in the form of modified couplings. One common framework for parameterizing new physics in diboson production is an effective Lagrangian [1] of the form:

$$\begin{aligned} \mathcal{L}^{WWX}\propto & {} \Big [ (1+\Delta g^X_1) (W^+_{\mu \nu }W^{-\mu } - W^{+\mu }W^-_{\mu \nu })X^\nu \\&+ (1+\Delta \kappa _X) W^+_\mu W^-_\nu X^{\mu \nu } + \frac{\lambda _X}{m_W^2} W^{+\nu }_\mu W_\nu ^{-\rho }X^\mu _\rho \Big ], \end{aligned}$$

where $X=Z$ or $\gamma$, $W^{\pm }_{\mu \nu }=\partial _\mu W^{\pm }_\nu - \partial _\nu W^{\pm }_\mu$, and $X_{\mu \nu }=\partial _\mu X_\nu - \partial _\nu X_\mu$. The six parameters $\lambda _X$, $\Delta \kappa _X$, and $\Delta g^X_1$ (hereafter called “aTGC parameters”) are all zero in the SM. The parameter $\Delta g^\gamma _1$ is zero because of EM gauge invariance, leaving five free aTGC parameters, which describe deviations of the triple gauge-boson couplings from their SM predictions. It is common to apply the so-called *LEP constraint* [76], which imposes ${SU}(2)\times U(1)$ gauge invariance, and which reduces the number of independent aTGC parameters to three, by introducing the following constraints: $\lambda _\gamma =\lambda _Z$ and $\Delta g^Z_1=\Delta \kappa _Z+ \Delta \kappa _\gamma \tan ^2\theta _W$, where $\theta _W$ is the weak mixing angle. Since aTGC parameters lead to violation of unitarity at high energies, form factors are often applied to them in order to ensure unitarity:

$$\begin{aligned} \alpha\rightarrow & {} \frac{\alpha }{\left( 1 + \frac{\hat{s}}{\Lambda _{\mathrm {FF}}^2}\right) ^2}, \end{aligned}$$

where $\alpha$ is one of the aTGC parameters, $\hat{s}$ is the square of the diboson invariant mass, and $\Lambda _{\mathrm {FF}}$ is the form factor’s energy scale.

An alternative framework for describing modifications of diboson production is an effective field theory (EFT) [77, 78] that is assumed to be valid below an energy scale $\Lambda$, and which introduces three CP-conserving dimension-six operators:

$$\begin{aligned} \mathcal{O}_{W}= & {} (D_\mu \Phi )^{\dagger } W^{\mu \nu } (D_\nu \Phi ), \\ \mathcal{O}_{B}= & {} (D_\mu \Phi )^{\dagger } B^{\mu \nu } (D_\nu \Phi ), \\ \mathcal{O}_{WWW}= & {} { Tr}[ W_{\mu \nu } W^{\nu \rho } W^{\mu }_{\rho }]. \end{aligned}$$

Here, $\Phi$ is the Higgs doublet field, $D_\mu$ is the covariant derivative, and $W^{\mu \nu }$ and $B^{\mu \nu }$ are the field strength tensors of the *W* and *B* gauge boson fields. The coefficients of these operators (EFT parameters), $c_W/\Lambda ^2$, $c_B/\Lambda ^2$, and $c_{WWW}/\Lambda ^2$, are zero in the SM and can be related to the LEP-constraint aTGC parameters as follows:

$$\begin{aligned} \frac{c_W}{\Lambda ^2}= & {} \frac{2}{m_Z^2}\Delta g^Z_1, \\ \frac{c_B}{\Lambda ^2}= & {} \frac{2}{m_W^2}\Delta \kappa _\gamma - \frac{2}{m_Z^2}\Delta g^Z_1, \\ \frac{c_{WWW}}{\Lambda ^2}= & {} \frac{2}{3 g^2 m_W^2} \lambda . \end{aligned}$$

This relation only holds if no form factor is applied to the aTGCs. The effect of aTGC/EFT parameters on the $H\rightarrow WW$ process is neglected.

The aTGC and EFT parameters both tend to increase the diboson cross-section at high $p_{\mathrm {T}}(V)$ and high invariant mass of the diboson system. Both the $WV \rightarrow \ell \nu \mathrm {j}\mathrm {j}$ channel and the $WV \rightarrow \ell \nu \mathrm {J}$ channel can be used to search for these BSM enhancements. The $WV \rightarrow \ell \nu \mathrm {J}$ channel, although currently less sensitive as a SM *WV* measurement, is expected to provide a higher sensitivity to the aTGC/EFT models, because of the better efficiency at high $p_{\mathrm {T}}(V)$. On the other hand, the $WV \rightarrow \ell \nu \mathrm {j}\mathrm {j}$ channel, where the SM *WV* measurement is clearly established, is useful as a complementary search channel that probes a different energy range.

In this analysis, the new-physics search uses signal regions with exactly the same event selection as the cross-section measurements, except that the $m_{\mathrm {j}\mathrm {j}}$ requirement is tightened to $65~\text {GeV}<m_{\mathrm {j}\mathrm {j}}<95~\text {GeV}$ in the $WV \rightarrow \ell \nu \mathrm {j}\mathrm {j}$ channel and the $m_{\mathrm {J}}$ requirement is tightened to $65~\text {GeV}<m_{\mathrm {J}}<95~\text {GeV}$ in the $WV \rightarrow \ell \nu \mathrm {J}$ channel. These tighter requirements lead to an increase in the signal-to-background ratio. In the $WV \rightarrow \ell \nu \mathrm {j}\mathrm {j}$ channel, events which fail the $m_{\mathrm {j}\mathrm {j}}$ requirement (i.e. $40~\text {GeV}<m_{\mathrm {j}\mathrm {j}}<65~\text {GeV}$ or $95~\text {GeV}<m_{\mathrm {j}\mathrm {j}}<200~\text {GeV}$) are put into a *sideband* control region. The *ZZ* background is neglected in the new-physics search, due to its very small expected contribution.

**Fig. 7:**
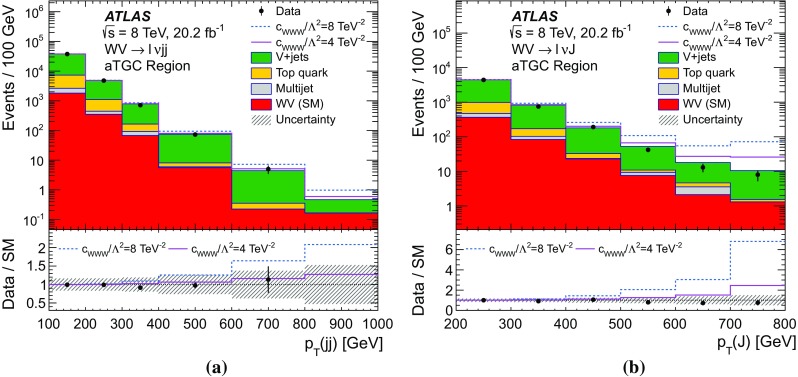
The observed **a** $p_{\mathrm {T}}(\mathrm {j}\mathrm {j})$ distribution in the $WV \rightarrow \ell \nu \mathrm {j}\mathrm {j}$ aTGC signal region, and **b** $p_{\mathrm {T}}(\mathrm {J})$ distribution in the $WV \rightarrow \ell \nu \mathrm {J}$ aTGC signal region, overlaid with the background and signal prediction. The expected BSM enhancements due to anomalous values of the EFT parameter $c_{WWW}/\Lambda ^2$ are also shown, for $c_{WWW}/\Lambda ^2=4~\mathrm {TeV^{-2}}$ and $c_{WWW}/\Lambda ^2=8~\mathrm {TeV^{-2}}$. The *hatched bands* indicate the systematic uncertainty in the SM prediction. The *histograms* are displayed with the binning that is used for the computation of the confidence intervals for the aTGC and EFT parameters. The last bin includes overflow

The search makes use of the $p_{\mathrm {T}}(\mathrm {j}\mathrm {j})$ ($WV \rightarrow \ell \nu \mathrm {j}\mathrm {j}$ channel) or $p_{\mathrm {T}}(\mathrm {J})$ ($WV \rightarrow \ell \nu \mathrm {J}$ channel) distribution. Hereafter, $p_{\mathrm {T}}(V_{\mathrm {rec}})$ is used to refer to both $p_{\mathrm {T}}(\mathrm {j}\mathrm {j})$ and $p_{\mathrm {T}}(\mathrm {J})$. The $p_{\mathrm {T}}(V_{\mathrm {rec}})$ distributions of the events in the signal regions are shown in Fig. 7. This figure also shows the expected enhancement at high $p_{\mathrm {T}}(V_{\mathrm {rec}})$ in the presence of different EFT parameter values. As can be seen from the figure, no significant deviation from the SM prediction is observed; therefore, $95\%$ confidence intervals are computed for the aTGC and EFT parameters.

The confidence intervals are computed from binned ML fits to the $p_{\mathrm {T}}(V_{\mathrm {rec}})$ distributions. The intervals are calculated using a frequentist Feldman–Cousins approach [79]. In the $WV \rightarrow \ell \nu \mathrm {j}\mathrm {j}$ channel, simultaneous fits to the $p_{\mathrm {T}}(V_{\mathrm {rec}})$ distributions in the signal region and sideband CR are used, while in the $WV \rightarrow \ell \nu \mathrm {J}$ channel, only the $p_{\mathrm {T}}(V_{\mathrm {rec}})$ distribution in the signal region is used. Since the $WV \rightarrow \ell \nu \mathrm {J}$ and $WV \rightarrow \ell \nu \mathrm {j}\mathrm {j}$ selections overlap, the confidence intervals are calculated separately for the $WV \rightarrow \ell \nu \mathrm {J}$ and $WV \rightarrow \ell \nu \mathrm {j}\mathrm {j}$ selections. In the fits, the SM *WV* and background predictions are allowed to vary within their uncertainties. The measured cross sections of Sect. 10 are consistent with theoretical SM *WV* predictions, but have large associated uncertainties; for this reason the theoretical prediction is used here. The systematic uncertainties in the normalizations and $p_{\mathrm {T}}(V_{\mathrm {rec}})$ shapes of the signal and backgrounds are accounted for through nuisance parameters. The systematic uncertainties that have the largest impact on the results are the jet-related uncertainties (in both channels) and the uncertainty from the limited size of the MC samples (in the $WV \rightarrow \ell \nu \mathrm {j}\mathrm {j}$ channel).

The observed 95% confidence intervals for the aTGC parameters are shown in Table 5, without applying the LEP constraint. The confidence intervals for a given aTGC parameter are computed while fixing the other aTGC parameters to zero. The confidence intervals are shown separately for the $WV \rightarrow \ell \nu \mathrm {j}\mathrm {j}$ and $WV \rightarrow \ell \nu \mathrm {J}$ selections, and the expected confidence intervals under the SM hypothesis are also shown for comparison. Confidence intervals for the aTGC parameters are shown for $\Lambda _{\mathrm {FF}}=5~\text {TeV}$ and for the case of no form factor (equivalent to $\Lambda _{\mathrm {FF}}=\infty$). The value of $\Lambda _{\mathrm {FF}}=5~\text {TeV}$ is chosen in order to ensure unitarity over the range of aTGC parameter values to which this analysis is sensitive [80].

The $WV \rightarrow \ell \nu \mathrm {J}$ selection has significantly better sensitivity to the aTGC parameters. No combination of the $WV \rightarrow \ell \nu \mathrm {j}\mathrm {j}$ and $WV \rightarrow \ell \nu \mathrm {J}$ constraints is performed, since it is expected that the $WV \rightarrow \ell \nu \mathrm {J}$ channel would dominate the combination. The sensitivity to the aTGC parameters in the $WV \rightarrow \ell \nu \mathrm {J}$ channel mainly comes from the $p_{\mathrm {T}}(V_{\mathrm {rec}})>600~\text {GeV}$ bins, whereas the sensitivity in the $WV \rightarrow \ell \nu \mathrm {j}\mathrm {j}$ channel mainly comes from the 300–600$~\text {GeV}$ bins. Since the $WV \rightarrow \ell \nu \mathrm {j}\mathrm {j}$ channel probes a lower $p_{\mathrm {T}}(V_{\mathrm {rec}})$ range, its sensitivity is less degraded by the form factors (which have a larger effect at higher $p_{\mathrm {T}}$) than the $WV \rightarrow \ell \nu \mathrm {J}$ channel.

In addition, the observed and expected confidence intervals for the aTGC parameters in the LEP-constraint scenario are given in Table 6 for $\Lambda _{\mathrm {FF}}=\infty$.

**Table 5:** The observed and expected 95% confidence intervals for the aTGC parameters without the LEP constraint. The confidence intervals are computed separately for the $WV \rightarrow \ell \nu \mathrm {j}\mathrm {j}$ and $WV \rightarrow \ell \nu \mathrm {J}$ channels, and are calculated both for $\Lambda _{\mathrm {FF}}=5~\text {TeV}$ and $\Lambda _{\mathrm {FF}}=\infty$ (i.e. no form factor). The confidence intervals for each parameter are calculated while fixing the other parameters to zero

Form factor	Parameter	Observed	Expected	Observed	Expected
		$WV \rightarrow \ell \nu \mathrm {j}\mathrm {j}$		$WV \rightarrow \ell \nu \mathrm {J}$	
	$\Delta g^Z_1$	[ $-0.039, 0.059$]	[ $-0.050, 0.066$]	[ $-0.033, 0.036$]	[ $-0.039, 0.042$]
	$\Delta \kappa _Z$	[ $-0.045, 0.063$]	[ $-0.060, 0.076$]	[ $-0.028, 0.030$]	[ $-0.033, 0.035$]
$\Lambda _{\mathrm {FF}}=\infty$	$\lambda _Z$	[ $-0.024, 0.024$]	[ $-0.029, 0.029$]	[ $-0.015, 0.015$]	[ $-0.017, 0.017$]
	$\Delta \kappa _\gamma$	[ $-0.099, 0.14$]	[ $-0.13, 0.17$]	[ $-0.058, 0.063$]	[ $-0.067, 0.073$]
	$\lambda _\gamma$	[ $-0.084, 0.084$]	[ $-0.10, 0.10$]	[ $-0.042, 0.041$]	[ $-0.049, 0.049$]
	$\Delta g^Z_1$	[ $-0.042, 0.064$]	[ $-0.055, 0.073$]	[ $-0.044, 0.048$]	[ $-0.051, 0.054$]
	$\Delta \kappa _Z$	[ $-0.047, 0.068$]	[ $-0.064, 0.083$]	[ $-0.037, 0.040$]	[ $-0.043, 0.047$]
$\Lambda _{\mathrm {FF}}=5~\text {TeV}$	$\lambda _Z$	[ $-0.026, 0.026$]	[ $-0.032, 0.032$]	[ $-0.020, 0.019$]	[ $-0.023, 0.022$]
	$\Delta \kappa _\gamma$	[ $-0.10, 0.15$]	[ $-0.14, 0.18$]	[ $-0.077, 0.084$]	[ $-0.089, 0.097$]
	$\lambda _\gamma$	[ $-0.089, 0.089$]	[ $-0.11, 0.11$]	[ $-0.056, 0.056$]	[ $-0.065, 0.065$]

**Table 6:** The observed and expected 95% confidence intervals for the aTGC parameters in the LEP-constraint scenario with $\Lambda _{\mathrm {FF}}=\infty$, computed separately for the $WV \rightarrow \ell \nu \mathrm {j}\mathrm {j}$ and $WV \rightarrow \ell \nu \mathrm {J}$ channels. The confidence intervals for each parameter are calculated while fixing the other parameters to zero

Parameter	Observed	Expected	Observed	Expected
	$WV \rightarrow \ell \nu \mathrm {j}\mathrm {j}$		$WV \rightarrow \ell \nu \mathrm {J}$	
$\Delta g^Z_1$	[ $-0.027, 0.045$]	[ $-0.036, 0.051$]	[ $-0.021, 0.024$]	[ $-0.024, 0.027$]
$\Delta \kappa _\gamma$	[ $-0.11, 0.13$]	[ $-0.15, 0.16$]	[ $-0.061, 0.064$]	[ $-0.071, 0.075$]
$\lambda _Z$= $\lambda _\gamma$	[ $-0.022, 0.022$]	[ $-0.027, 0.026$]	[ $-0.013, 0.013$]	[ $-0.015, 0.015$]

**Fig. 8:**
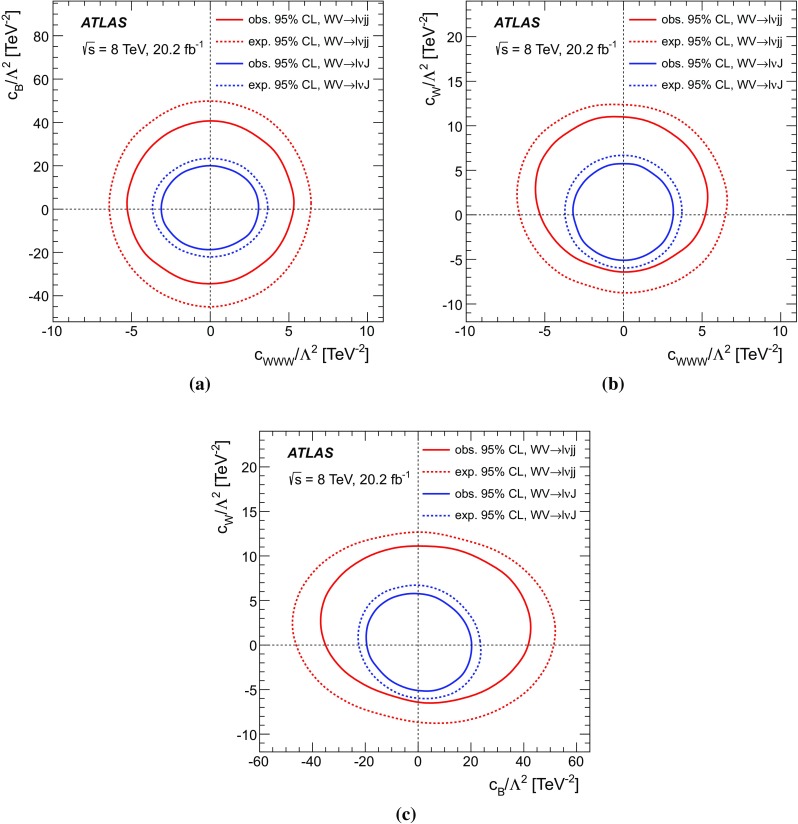
The 95% confidence-level regions for combinations of two EFT parameters. **a** $c_{WWW}/\Lambda ^2$ and $c_B/\Lambda ^2$, **b** $c_{WWW}/\Lambda ^2$ and $c_W/\Lambda ^2$, **c** $c_B/\Lambda ^2$ and $c_W/\Lambda ^2$. The expected and observed confidence regions are shown for the $WV \rightarrow \ell \nu \mathrm {j}\mathrm {j}$ channel (*outer contours*) and the $WV \rightarrow \ell \nu \mathrm {J}$ channel (*inner contours*). When computing the confidence regions for two parameters, the third EFT parameter is held fixed to zero

The observed and expected confidence intervals for the EFT parameters are shown in Table 7, separately for the $WV \rightarrow \ell \nu \mathrm {j}\mathrm {j}$ and $WV \rightarrow \ell \nu \mathrm {J}$ selections. Confidence regions for combinations of two EFT parameters are shown in Fig. 8; for each combination the third EFT parameter is held fixed to zero. Although the constraints from the $WV \rightarrow \ell \nu \mathrm {j}\mathrm {j}$ channel are less stringent than those from the $WV \rightarrow \ell \nu \mathrm {J}$ channel, they probe a complementary phase space. The sensitivity of the $WV \rightarrow \ell \nu \mathrm {J}$ channel is similar to the most sensitive previous analyses to publish constraints on these parameters [3, 5, 6, 22]. The $WV \rightarrow \ell \nu \mathrm {J}$ channel probes a similar phase space to Ref. [22]; these analyses benefit from their ability to reconstruct high-$p_{\mathrm {T}}$ $V \rightarrow q q^{\prime }$ decays.

**Table 7:** The observed and expected 95% confidence intervals for the EFT parameters. The parameters are given in units of $\mathrm {TeV^{-2}}$. The confidence intervals for each parameter are calculated while fixing the other parameters to zero

Parameter	Observed ($\mathrm {TeV^{-2}}$)	Expected ($\mathrm {TeV^{-2}}$)	Observed ($\mathrm {TeV^{-2}}$)	Expected ($\mathrm {TeV^{-2}}$)
	$WV \rightarrow \ell \nu \mathrm {j}\mathrm {j}$		$WV \rightarrow \ell \nu \mathrm {J}$	
$c_{WWW}/\Lambda ^2$	[ $-5.3, 5.3$]	[ $-6.4, 6.3$]	[ $-3.1, 3.1$]	[ $-3.6, 3.6$]
$c_B/\Lambda ^2$	[ $-36, 43$]	[ $-45, 51$]	[ $-19, 20$]	[ $-22, 23$]
$c_W/\Lambda ^2$	[ $-6.4, 11$]	[ $-8.7, 13$]	[ $-5.1, 5.8$]	[ $-6.0, 6.7$]

## Conclusion

The production of $WV \rightarrow \ell \nu q q^{\prime }$, with *V* being a *W* or *Z* boson, is measured using $20.2~\text{ fb }^{-1}$ of *pp* collisions at $8~\text {TeV}$ at the LHC with the ATLAS detector. The measurements focus on *WV* production where the bosons have large transverse momentum. Fiducial cross-sections for the $WV \rightarrow \ell \nu q q^{\prime }$ process are measured in two different, but partially overlapping, phase spaces.

The first phase space, denoted $WV \rightarrow \ell \nu \mathrm {j}\mathrm {j}$, targets a hadronically decaying *V* boson whose decay products can be distinguished as two $R=0.4$ jets. In this phase space, the $WV \rightarrow \ell \nu q q^{\prime }$ process is measured with a significance of $4.5\sigma$, and the fiducial cross-section is measured to be $209 \pm 28 (\text {stat}) \pm 45 (\text {syst})~\text {fb}$, in agreement with the MC@NLO prediction of $225 \pm 13~\text {fb}$.

The second phase space, denoted $WV \rightarrow \ell \nu \mathrm {J}$, contains a single $R=1.0$ jet consistent with the collimated decay products of a high-$p_{\mathrm {T}}$ *V* boson. The *WV* process is measured with a significance of $1.3\sigma$ in this phase space. The fiducial cross-section for this phase space is measured to be $30 \pm 11 (\text {stat}) \pm 22 (\text {syst})~\text {fb}$, consistent with the MC@NLO prediction of $58 \pm 15~\text {fb}$.

The events are also used to search for new physics modifying triple gauge-boson vertices, which could lead to enhancements of the cross-section at high $p_{\mathrm {T}}$ of the bosons. No evidence is found for new physics, and $95\%$ confidence intervals are computed for anomalous coupling parameters. The constraints on new physics are also interpreted in terms of an effective field theory. The $WV \rightarrow \ell \nu \mathrm {J}$ channel is found to be significantly more sensitive to the new-physics parameters than the $WV \rightarrow \ell \nu \mathrm {j}\mathrm {j}$ channel, which demonstrates the power of large-radius jet substructure techniques. The constraints from this analysis on the new physics parameters are comparable to the previous most stringent constraints from other diboson analyses.

## Footnotes

1 ATLAS uses a right-handed coordinate system with its origin at the nominal interaction point (IP) in the centre of the detector and the *z*-axis along the beam pipe. The *x*-axis points from the IP to the centre of the LHC ring, and the *y*-axis points upward. Cylindrical coordinates $(r,\phi )$ are used in the transverse plane, $\phi$ being the azimuthal angle around the *z*-axis. The pseudorapidity is defined in terms of the polar angle $\theta$ as $\eta =-\ln \tan (\theta /2)$.

2 The transverse mass is defined as $m_{\mathrm {T}}\equiv \sqrt{(E_{\text {T}}^{\text {miss}} +p_{\mathrm {T}}(\ell ))^2 - |\vec {E}_{\text {T}}^{\text {miss}} +\vec {p_{\mathrm {T}}}(\ell )|^2}$, where $p_{\mathrm {T}}(\ell )$ is the transverse momentum of the lepton candidate.

3 The significance is calculated based on the profile-likelihood ratio of the background-only and signal-and-background hypotheses. This ratio is converted to a significance using the asymptotic approximation [74].
